# Retinoic Acid Excess Impairs Amelogenesis Inducing Enamel Defects

**DOI:** 10.3389/fphys.2016.00673

**Published:** 2017-01-06

**Authors:** Supawich Morkmued, Virginie Laugel-Haushalter, Eric Mathieu, Brigitte Schuhbaur, Joseph Hemmerlé, Pascal Dollé, Agnès Bloch-Zupan, Karen Niederreither

**Affiliations:** ^1^Developmental Biology and Stem Cells Department, Institute of Genetics and Molecular and Cellular Biology (IGBMC) Illkirch, France; ^2^Centre National de la Recherche Scientifique, UMR 7104 Illkirch, France; ^3^Institut National de la Santé et de la Recherche Médicale, U 964 Illkirch, France; ^4^Université de Strasbourg Illkirch, France; ^5^Pediatrics Department, Faculty of Dentistry, Khon Kaen University Khon Kaen, Thailand; ^6^Université de Strasbourg, INSERM UMR_1121, Biomaterials and Bioengineering Strasbourg, France; ^7^Faculté de Chirurgie Dentaire, Université de Strasbourg Strasbourg, France; ^8^Faculté de Médecine, Fédération de Médecine Translationnelle de Strasbourg, Université de Strasbourg Strasbourg, France; ^9^Hôpitaux Universitaires de Strasbourg, Pôle de Médecine et Chirurgie Bucco-Dentaires, Centre de Référence des Manifestations Odontologiques des Maladies Rares, CRMR Strasbourg, France; ^10^Eastman Dental Institute, University College London London, UK

**Keywords:** retinoids, tooth, enamel, RNA-seq, mouse models, *enamelin*

## Abstract

Abnormalities of enamel matrix proteins deposition, mineralization, or degradation during tooth development are responsible for a spectrum of either genetic diseases termed *Amelogenesis imperfecta* or acquired enamel defects. To assess if environmental/nutritional factors can exacerbate enamel defects, we investigated the role of the active form of vitamin A, retinoic acid (RA). Robust expression of RA-degrading enzymes *Cyp26b1* and *Cyp26c1* in developing murine teeth suggested RA excess would reduce tooth hard tissue mineralization, adversely affecting enamel. We employed a protocol where RA was supplied to pregnant mice as a food supplement, at a concentration estimated to result in moderate elevations in serum RA levels. This supplementation led to severe enamel defects in adult mice born from pregnant dams, with most severe alterations observed for treatments from embryonic day (E)12.5 to E16.5. We identified the enamel matrix proteins *enamelin* (*Enam*), *ameloblastin* (*Ambn*), and *odontogenic ameloblast-associated protein* (*Odam*) as target genes affected by excess RA, exhibiting mRNA reductions of over 20-fold in lower incisors at E16.5. RA treatments also affected bone formation, reducing mineralization. Accordingly, craniofacial ossification was drastically reduced after 2 days of treatment (E14.5). Massive RNA-sequencing (RNA-seq) was performed on E14.5 and E16.5 lower incisors. Reductions in *Runx2* (a key transcriptional regulator of bone and enamel differentiation) and its targets were observed at E14.5 in RA-exposed embryos. RNA-seq analysis further indicated that bone growth factors, extracellular matrix, and calcium homeostasis were perturbed. Genes mutated in human AI (*ENAM, AMBN, AMELX, AMTN, KLK4*) were reduced in expression at E16.5. Our observations support a model in which elevated RA signaling at fetal stages affects dental cell lineages. Thereafter enamel protein production is impaired, leading to permanent enamel alterations.

## Introduction

Enamel formation is a unique biomineralization process involving a highly organized matrix protein scaffold deposition and degradation, leading to hydroxyapatite crystal nucleation generating a dense and tightly aligned network of hydroxyapatite crystals. Mature enamel is the body's hardest tissue. Ameloblasts are cells of ectodermal origin responsible for enamel development. These cells secrete enamel proteins, which are required for correct mineralization and structural maturation. Enamel proteins self-assemble and provide a matrix organization that aligns the thin ribbons of calcium phosphate deposited during enamel appositional growth. Enamel formation is characterized by inductive, secretory, and maturation stages. During the inductive stage, the inner enamel epithelium begins to differentiate. Then at the secretory stage, polarized differentiated ameloblasts release enamel proteins, contributing to the enamel matrix (Bei, 2009). Finally at the maturation stage, ameloblasts absorb water and organic matrix. This dehydration allows dense crystal deposition. Mature enamel is thus extremely strong, because of the density and fine organization of its crystal layers (Bei, 2009; Simmer et al., 2010).

*Amelogenesis imperfecta* (AI) refers to a group of rare genetic diseases presenting with defects in enamel formation either as isolated trait, or in association with other symptoms. Patient cases of AI are classified into hypoplastic, hypomineralized, or hypomaturation categories based on enamel quantitative or qualitative defects, i.e., thickness, hardness, and/or color. To date, mutations in over 30 genes are associated with non-syndromic or syndromic AI (Bloch-Zupan et al., 2012). These diseases can be recapitulated in several mouse models. For example, hypoplastic or aplastic enamel deficiencies are produced by *amelogenin (Amel), ameloblastin* (*Ambn*), and *enamelin* (*Enam*) mutations, recapitulating defects in patients (Gibson et al., 2001; Fukumoto et al., 2004; Masuya et al., 2005; Hu et al., 2008, 2014). Studies in mouse models have contributed to understanding how defective ameloblast protein secretion contributes to these diseases. For a given genetic defect, interfamilial, intrafamilial, and individual intraoral variations in phenotype severity are often seen, suggesting that environmental factors come into play. Indeed, ameloblasts are highly sensitive to their environment (Simmer et al., 2010). Body stressors including high fever (Ryynänen et al., 2014), excess fluoride (Yang et al., 2015), and endocrine disrupters such as bisphenol A disrupt ameloblast function (Jedeon et al., 2014). It is likely that a variety of factors contribute to clinical heterogeneity found in AI and to acquired enamel defects such as molar incisor hypomineralization (MIH) or hypomineralized second primary molars (HSPM; Alaluusua, 2010; Jeremias et al., 2013). Our objective is to discover novel factors initiating and influencing enamel regulatory networks, with the aim of designing new strategies to alleviate dental defects. To further characterize nutritional and environmental factors regulating enamel formation, we have focused on the role of retinoic acid (RA), the main active form of vitamin A that plays key roles during vertebrate development (Niederreither and Dollé, 2008). RA is the ligand for nuclear receptors (RARα, β, and γ), which bind as heterodimers with RXRs to DNA regulatory elements termed RA-response elements (RAREs, Rochette-Egly and Germain, 2009). Through this mechanism, RA regulates expression of various target genes (Balmer and Blomhoff, 2002). RA distribution within embryonic tissues is tightly controlled through an interplay between enzymes involved in its synthesis (mainly retinol dehydrogenase 10 and retinaldehyde dehydrogenases [Raldh]1, 2, and 3) and catabolism (Cyp26A1, B1, and C1). As both RA deficiency and excess results in diverse developmental defects, the distribution of active retinoid signaling requires tight regulation to limit potent adverse effects (Rhinn and Dollé, 2012).

Expression of RA receptors, synthesizing, and catabolizing enzymes has been detected in the developing teeth (Bloch-Zupan et al., 1994; Berkovitz et al., 2001; Cammas et al., 2007). Severe dietary vitamin A deficiency in rats leads to hypoplastic enamel, enamel organ metaplasia, dentine dysplasia, and delayed tooth eruption (Mellanby, 1941; Punyasingh et al., 1984; McDowell et al., 1987). Hypervitaminosis A during rodent pregnancy induces exencephaly and craniofacial malformations, along with tooth fusions and supernumerary incisors (Knudsen, 1967 and references therein). These effects may reveal an evolutionary role of RA signaling in the posterior pharyngeal region controlling tooth number (Seritrakul et al., 2012). In culture, RA excess can retard molar growth (Mark et al., 1992), reduce ameloblast differentiation (Kronmiller et al., 1992), and diminish tooth alkaline phosphatase production (Jones et al., 2008). Retinoids may also regulate incisor cervical loop maturation, increasing mitosis and laminin gene expression (Bloch-Zupan et al., 1994).

*In vivo* models to substantiate RA actions in tooth development are lacking. Interestingly, though, *Cyp26b1* knockout mice show a defect in maxillary bone compaction around upper incisors (Maclean et al., 2009). Observing that the fetal tooth has robust expression of *Cyp26b1* and *Cyp26c1* RA-degrading enzymes, we hypothesized that *in vivo*, conditions of RA excess may have adverse effects on osteoblast and ameloblast growth regulatory networks. We report that mice born from dams exposed to RA during mid-late pregnancy using a food-based supplementation suffer adult-stage enamel hypoplasia. Effects were strongest when treatments began at the tooth dental lamina-placode stage (E12.5), and continued until early bell developmental stages. Reductions of *Enam, Ambn*, and *Odam* mRNA expression in E16.5 lower incisors were observed. High throughput RNA sequencing (RNA-seq) analysis of lower incisors revealed that RA excess perturbs neural crest lineage determinants and pro-ossification growth factor and transcription networks. Combinatorial changes in collagen, extracellular matrix, and calcium homeostasis genes occur at E14.5, followed by a decrease at E16.5 of transcripts encoding pre-ameloblast secretory-stage proteins. These alterations in gene expression are observed several days before the ameloblast lineage begins to differentiate. Retinoid excess targets fetal odontoblasts, along with a range of epithelial enamel protein targets. Our data provides potential avenues through which environmental and nutritional changes may alter the penetrance and expressivity of human enamel defects such as AI or MIH.

## Materials and methods

### Ethics statement

All animals were maintained and manipulated under animal protocols in agreement with the French Ministry of Agriculture guidelines for use of laboratory animals (IGBMC protocol 2012–097) and with NIH guidelines, provided in the Guide for the Care and Use of Laboratory Animals. CD1 mice were purchased from Charles River, France. All-*trans*-RA (Sigma) suspended in ethanol (5 mg/mL) was mixed into 50 mL water and 50 g powdered food to a final concentration of 0.4 mg/g food. The RA-containing food mixture (protected from light) was left in the cage for the mice to feed *ad libitum* and changed every day. Control groups consisted of CD1 mice given a matching food treatment, but with no added RA. Equal numbers of RA-treated samples vs. controls were randomly assigned to treatment or control groups.

### *In situ* hybridization, beta-galactosidase (X-gal) staining, and skeletal analysis

*In situ* hybridization was performed using digoxigenin-labeled RNA probes on 200 μM vibratome sections of paraformaldehyde-fixed embryos, which were processed using an Intavis InSituPro robot, as described in detail on the website http://empress.har.mrc.ac.uk/browser/ (Gene Expression section). To analyze patterns of RA-response, we used the *RARE-hsp68-lacZ* reporter transgenic line (Rossant et al., 1991). At least 20 randomized fetal samples from control and matching RA-treated samples were used to test each probe. All expression studies were confirmed in at least 3 independent experiments. X-gal assays were performed on 200 μm vibratome sections. Whole-mount fetal alizarin red/alcian blue staining was carried out as described in http://empress.har.mrc.ac.uk/browser/ (Bone, Cartilage, Arthritis, Osteoporosis section).

### Real-time quantitative RT-PCR

RT-PCR assays were performed in duplicate on 3 RNA samples for control or RA-treated incisors dissected at E14.5 and E16.5. Total RNA (1 μg) was subjected to real-time RT-PCR using SYBR Green Reagents (Qiagen). RNA was extracted using the RNeasy Micro-kit. Oligo-dT primed cDNAs were generated using the Superscript II kit (Invitrogen). The incorporation of SYBR Green into the PCR products was monitored in real-time with a Roche 480 LightCycler. Sequences of primers are given in supplemental Table S1. Target genes were normalized relative to the glyceraldehyde-3-phosphate dehydrogenase (*Gapdh*) housekeeping gene.

### X-ray microtomography

Seven week-old mice were analyzed by X-ray micro-computed tomography (micro-CT) using a Quantum FX micro-CT *in vivo* Imaging System (Caliper Life Sciences), which operates at 80 kV and 160 μA, with high-resolution at 10–80 μm pixel size, to assay skull and tooth morphology. Data reconstructions were performed with the Analyze software (v 11.0; Biomedical Imaging Resource, Mayo Clinic, Rochester, MN).

### Scanning electron microscopy

The lower incisors of 7 week-old control and RA-treated mice were dissected out of the alveolar bone, rinsed, dehydrated in a graded series of ethanol, and then transferred in a propylene oxide/epon resin (v/v) solution. After embedding the teeth in Epon 812 (Euromedex, Souffelweyersheim, France), they were sectioned sagittally and polished with diamond pastes (Escil, Chassieu, France). The embedded half incisors were etched with a 20% (m/v) citric acid solution for 2 min, rinsed with distilled water, dehydrated in a graded series of ethanol and dried at room temperature. The samples were then coated with a gold-palladium alloy using a HUMMER JR sputtering device (Technics, CA, USA) before performing scanning electron microscopy with a Quanta 250 ESEM (FEI Company, Eindhoven, The Netherlands) with an accelerating voltage of the electrons of 5 kV.

### RNA sequencing

Total RNA was extracted in quadruplicate from lower incisors at E14.5 and 16.5 (2 days or 4 days after RA treatment) and respective controls. The mRNA-seq libraries were prepared as detailed in the Illumina protocol (supplemental Experimental Procedures). Sequence reads mapped to the mouse reference genome mm10/NCBI37 using Tophat. Only uniquely aligned reads were retained for further analysis. Quantification of gene expression was performed with HTSeq-0.6.1. (see http://www-huber.embl.de/users/anders/HTSeq/doc/overview.html). For each transcript the resulting reads per kilobase of exon model per million mapped reads (RPKM) were converted to raw read counts, which were then added for each gene locus. Data normalization was performed as described (Anders and Huber, 2010) and resolved using the DESeq Bioconductor package. Resulting *p*-values were adjusted for multiple testing, according to Benjamini and Hochberg (1995). Regulated transcripts with an RPKM of >2, an adjusted *p* < 0.050, and a log2 fold change of >0.3 and < −0.3 at E14.5 and >0.5 and < −0.5 at E16.5 were considered.

## Results

**Figure 1:**
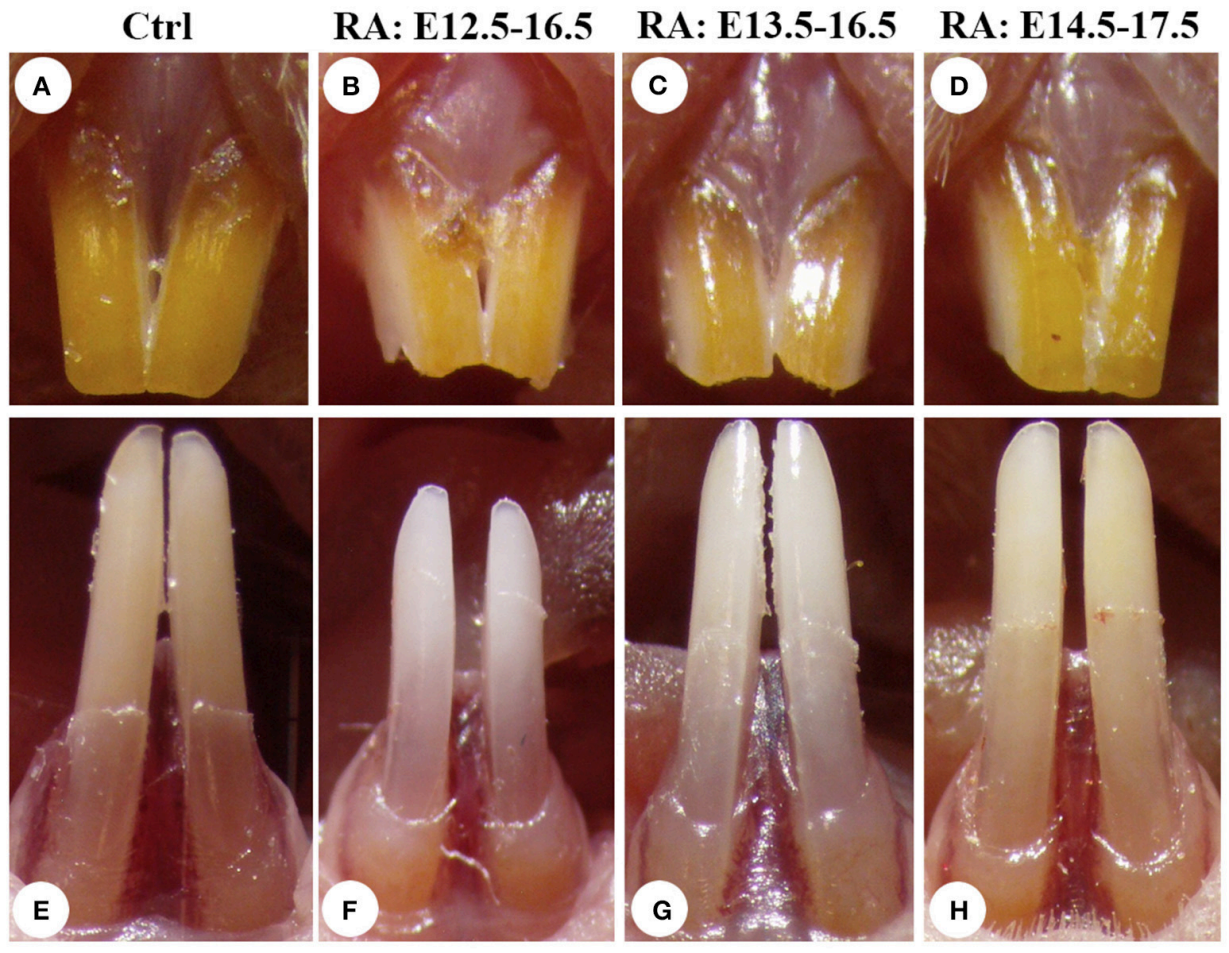
**RA excess during pregnancy produces stage-specific whitening and size reductions of mouse incisors**. Enamel of the upper **(A)** and lower **(E)** incisors of 7 weeks-old CD1 control (non-RA treated) mice has a characteristic yellow/orange color, which is consistently darker in the upper incisor. Retinoic acid treatment from E12.5 to 16.5 reduces incisor length (by ~20%) and lightens enamel color **(B,F)**, suggesting reduced iron accumulation typical of murine models of hypoplastic enamel formation. When RA treatment begins at E13.5 **(C,G)** or E14.5 **(D,H)**, incisor length reductions and lightening of incisor color are progressively less severe.

To analyze RA-dependent tooth alterations, we employed a protocol where RA added to the food supply was administered to pregnant CD1 mice (Niederreither et al., 2002), at a concentration of 0.4 mg/g food beginning at E12.5 or later. The treatment period began after craniofacial neural crest migration into the head was complete (Minoux and Rijli, 2010), avoiding earlier stage lethality due to exencephaly. In another study, HPLC analysis carried out after similar RA treatment at 0.1 mg/g food showed that serum RA levels were moderately increased (~20%) compared with untreated controls (Mic et al., 2003). Treated dams bore litters with 50% lethality, typically with 5–7 pups. Incisors in both groups erupted at the same age. Once the pups reached adulthood (4–7 weeks-old), we compared 100 randomized control and RA-treated groups macroscopically for dental defects. The labial side of rodent incisor (analog of the crown and covered with enamel) normally has a yellow/orange pigmentation, due to iron present at a net weight of about 0.03% in enamel (Pindborg, 1953). It gives mouse teeth a characteristic color, which is a dark orange in the upper incisors (Figures 1A,E). When RA treatments were performed from E12.5 to 16.5, they were found to produce a chalky lightening and length reduction of incisors, changes more pronounced in lower incisors (Figures 1B,F). The whiter color and less shiny surface may reflect reduced enamel thickness typical of mouse enamel hypoplasia models (Gibson et al., 2001; Masuya et al., 2005; Hu et al., 2014). Treatments performed at later stages (E13.5–16.5: Figures 1C,G, or E14.5–17.5: Figures 1D,H) had less severe effects on enamel, suggesting RA has early roles in the oral epithelium starting at the placode stage of tooth initiation.

**Figure 2:**
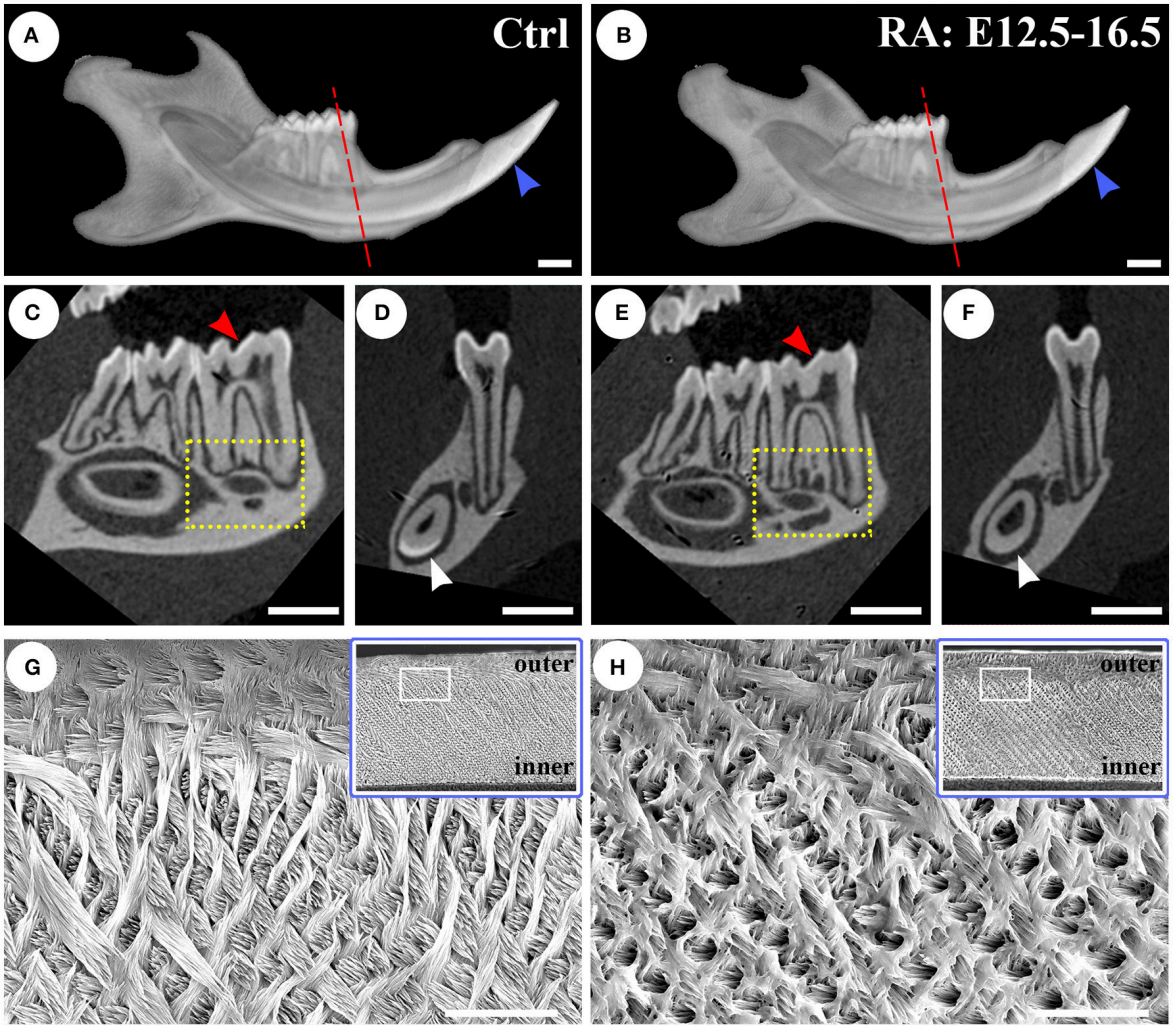
**Micro-CT and SEM imaging of 7 weeks-old control (A,C,D,G)** and RA-treated **(B,E,F,H)** mice. **(A)** Shows the typical length of a control incisor, which is longer and exhibits an opaque whiter region reflecting high density where enamel is present. Comparing the E12.5–16.5 RA-treated mouse **(B)**, incisor length and enamel density are reduced throughout the tooth. Optical sections in a sagittal plane reveal regions with less surface enamel on lower molars of the treated mouse (arrowheads in **C,E**). Optical cross-sections of the first molar and lower incisor (dotted lines in **A,B**) show reduced density of enamel in the lower incisor (arrowheads in **D,F**). An increased porosity of the alveolar bone is observed below the molars in the RA-treated mouse (compare boxed areas in **C,E**). Scale bars: 1 mm. **(G,H)** Are SEM images from the central longitudinal plane of fully-formed lower incisor enamel from control and RA-treated samples (arrowheads in **A,B)**. The enamel of control presented a constant thickness and a clear decussating prism pattern **(G)**. A reduced mineralization and impaired organization (especially in the outer enamel) is observed in RA-treated samples **(H)**. The area from which SEM views are taken is shown in the insert box. Scale bars: 10 μm.

Samples shown in Figures 1A,E (WT) and B,F (RA-treated) were analyzed by X-ray micro-computed tomography (micro-CT). This analysis confirmed lower incisor shortening (Figures 2A,B; Figure S1). Optical sections revealed a reduction in enamel mineral density and thickness (molars in Figures 2C,E; incisors in Figures 2D,F). Alveolar bone at the level of the molars showed greater porosity in the RA-treated animals (boxes in Figures 2C,E; Figure S2). Although retinoid gradients shape pharyngeal tooth evolution (Seritrakul et al., 2012), our micro-CT analysis revealed normal molar eruption and cusp morphology (Figure S3), as predicted because RA treatments are initiated at E12.5, after neural crest has completed its migration into the jaw. This analysis also revealed no evident signs of dental attrition from both groups. Figure 2G shows scanning electron micrographs (SEM) of enamel prisms of control lower incisor, exhibiting a well-organized decussating pattern. This was disrupted following RA treatment. The most outer enamel was less mineralized when compared with the control tooth, as outer enamel appeared darker in RA-treated animals. Enamel rods of RA-supplemented animals were less densely packed, and as a consequence, areas normally filled with interprismatic enamel seemed empty and showed holes-like pattern (Figure 2H), similar to *Enam* haploinsufficent mice (Hu et al., 2014).

**Figure 3:**
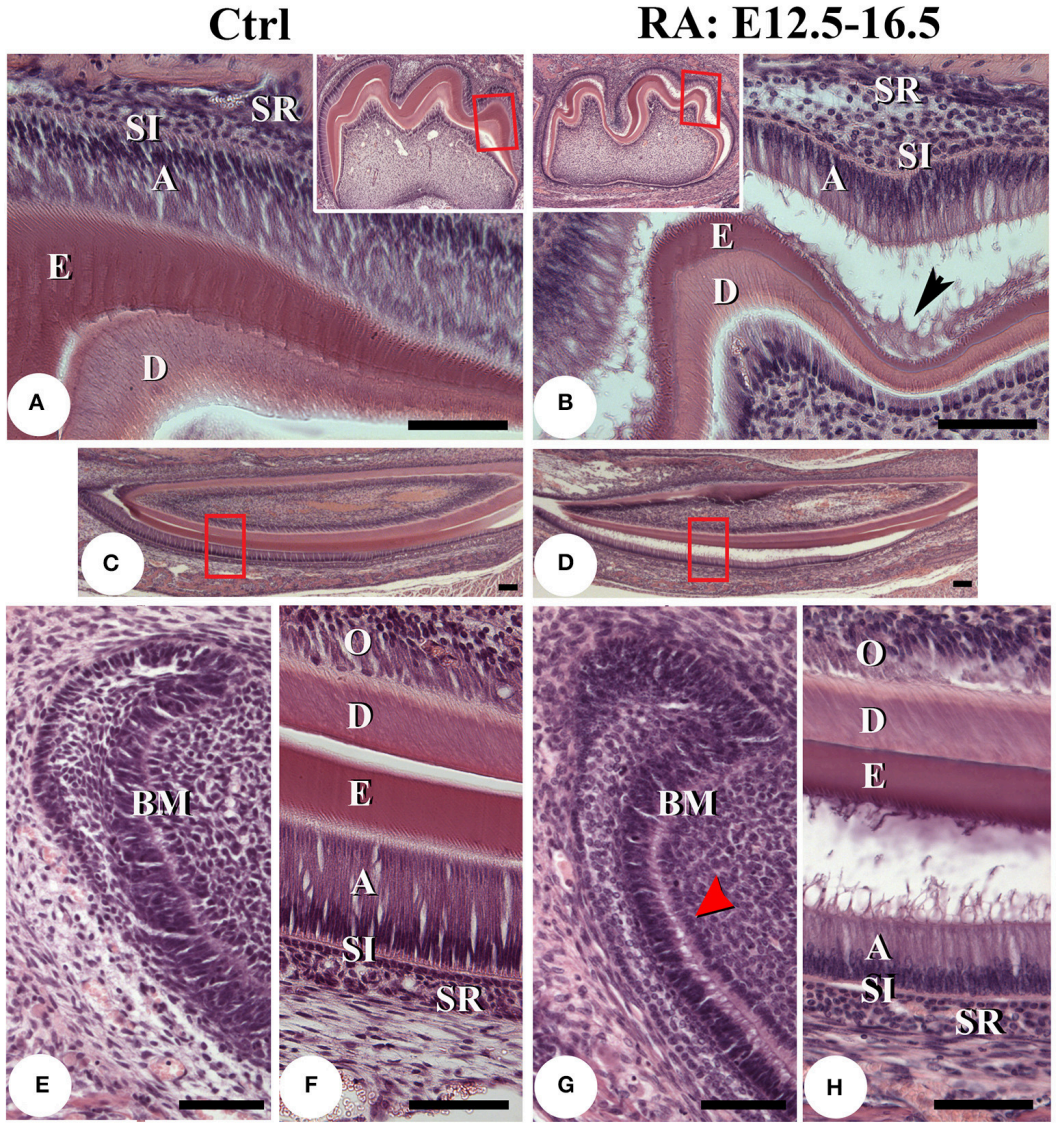
**Histological analysis of post-natal day 7 teeth**. Hematoxylin and eosin stained sagittal sections of lower first molars of control **(A)** and E12.5–16.5 RA-treated **(B)** mice. The boxed areas in low-magnification insert panels are shown in detail. A black arrowhead in **B** points to cell debris accumulating between separated tissue layers. Lower incisors of untreated **(C,E,F)** and RA-treated **(D,G,H)** mice. **F**, **H** are higher magnification views of the boxed areas in **C,D**. Comparing the cervical loop of untreated **(E)** with RA-treated mice **(G)**, it appears retinoid excess may disrupt the basement membrane (**G**, red arrowhead), potentially leading to ameloblast detachment **(B,D,H)**. The secretory stage ameloblast layer is also slightly thinner and enamel thickness reduced after RA treatment **(B,D,H)**. Scale bars: 100 μm. Abbreviations: A, ameloblasts; BM, basement membrane; D, dentin; E, enamel; O, odontoblasts; SI, stratum intermedium; SR, stellate reticulum.

Histological analysis of 1-week old mice after hematoxylin-eosin staining confirmed that RA treatments produced a shorter, smaller, and disorganized layer of ameloblasts in both molars (Figures 3A,B) and incisors (Figures 3C–H). This was clearly seen in the secretory zone, where ameloblasts displayed disrupted epithelial sheet organization (Figure 3H). In all treated samples, ameloblast adhesion to enamel was impaired (Figures 3B,D,G,H). This was first seen histologically as a detachment of the basement membrane, likely causing preameloblast separation from forming odontoblasts (Figure 3G, red arrowhead). Non-cellular organic material was present between layers (Figure 3B, black arrowhead). The lower incisor secretory zone enamel layer was slightly thinner (Figure 3H), suggesting RA treatment delayed enamel maturation and/or reduced overall mineralization.

**Figure 4:**
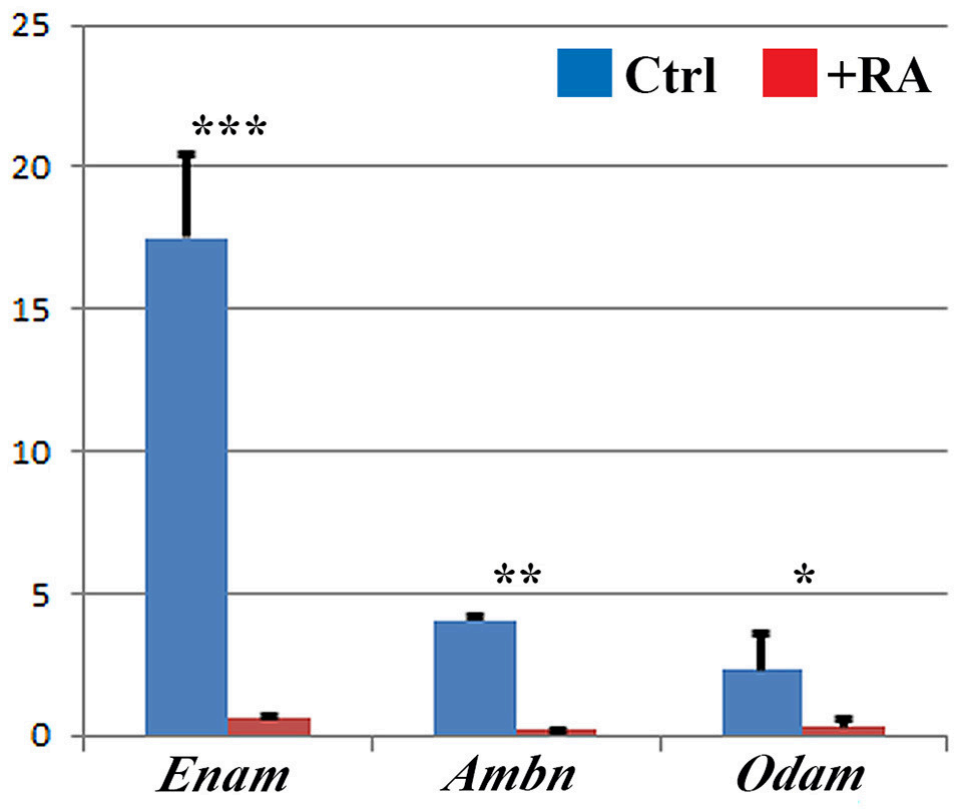
**Real-time RT-PCR analysis shows reductions in secretory-stage enamel gene expression**. *Enam, Ambn*, and *Odam* mRNA levels in lower incisor samples of E16.5 control mice (blue) vs. E12.5–16.5 RA-treated mice. Target genes were normalized relative to *Gapdh*. Mean (*SD*). Student *t*-test: ^***^p < 0.001, ^**^p < 0.010, ^*^p < 0.050.

Enamel matrix proteins are secreted by ameloblasts and form a matrix directing enamel mineral deposition. Among these proteins, amelogenin is the most abundant (Eastoe, 1979). Enamel fails to form or is hypoplastic in amelogenin-deficient mice (Gibson et al., 2001), and in *Enam*- or *Ambn*-null mutant mice (Fukumoto et al., 2004; Masuya et al., 2005; Hu et al., 2014). Our real-time RT-PCR analysis of E16.5 lower incisors following E12.5–16.5 RA treatment revealed up to 20-fold reductions in *Enam, Ambn*, and *Odam* mRNAs (Figure 4). Notably, no RA-induced alterations in *Enam, Ambn*, or *Odam* were observed at E14.5 (data not shown).

**Figure 5:**
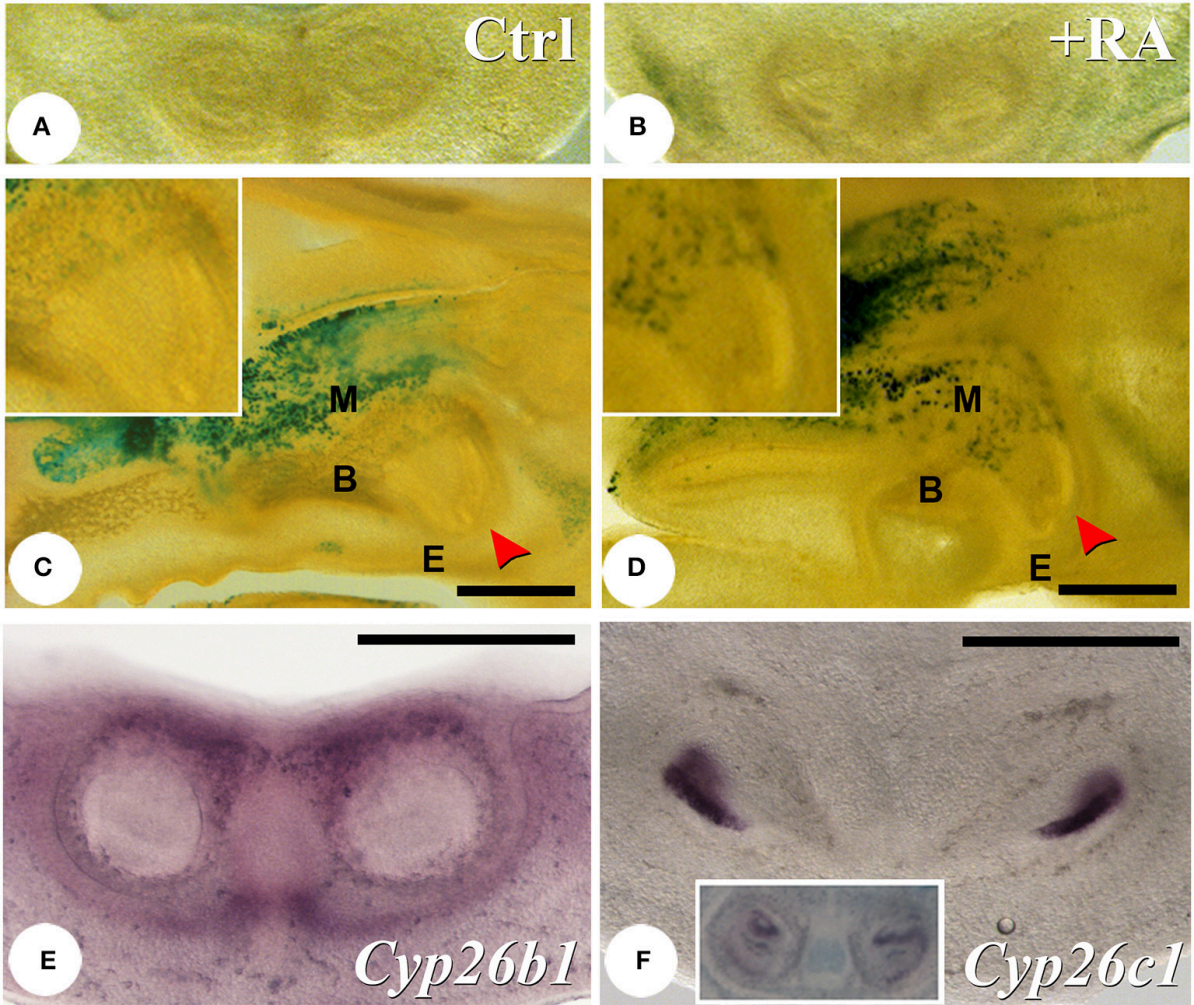
**Regions of RA activity and expression of RA-catabolizing enzymes in the developing teeth**. X-gal analysis of RARE-hsp68-*lacZ* reporter transgene activation in E14.5 **(A,B)** and E16.5 **(C,D)** untreated and RA-treated (E12.5–16.5) mice. At E14.5 in untreated mice, transgene activity can be observed surrounding the tooth germ, and this activity is increased in treated mice. At E16.5, X-gal activity is detected in alveolar bone adjacent to upper **(C,D)** and lower (data not shown) incisors (arrowhead). After RA treatment, reporter activity is increased in alveolar bone, but is never seen in developing teeth (compare high magnification insert panels in **C,D**). At E13.5, *Cyp26b1* is expressed in mesenchyme surrounding the lower incisors **(E)**. At E14.5 a pronounced expression of *Cyp26c1* is seen in the enamel organ **(F)**, whereas *Cyp26a1* is expressed in the dental papilla epithelium (**F**, insert). Scale bars: 500 μm. Abbreviations: B, bone; E, epithelium; M, mesenchyme.

To assess if reduced enamel protein expression was linked to ectopic activation of RA signaling, we used RARE-hsp68-*lacZ* transgenic mice as a reporter for RA activity (Rossant et al., 1991). No expression was seen in the tooth germ areas at E13.5 (data not shown), although activity was found in the mandible next to the tooth germs at E14.5 (Figures 5A,B). This retinoid reporter transgene exhibited low level of expression in both mandible and maxilla adjacent to the growing incisors and molars in E16.5 untreated fetuses (Figure 5C and data not shown). In E16.5 fetuses following RA treatment from E12.5 to 16.5, domains of RARE-*lacZ* activity broadly extended into alveolar bone and surrounding mesenchyme, but appeared absent from enamel organ and dental ectomesenchyme (Figure 5D and data not shown). Note that very low levels of RA signaling may not be detected by such a reporter transgene. Proper control of RA levels is necessary for early neural crest patterning, but RA deficiency does not alter first branchial arch formation (Niederreither et al., 2000). To explore why RA activity was absent from most tissues of the tooth buds, we examined expression of CYP26 family cytochrome P450 enzymes specifically involved in RA catabolism. At E13.5, *Cyp26b1* expression (Figure 5E) was found to be prominent in mesenchyme surrounding the forming incisors. At E14.5, *Cyp26c1* (Figure 5F) was prominently expressed in the enamel organ, whereas a low amount of *Cyp26a1* transcripts was seen in the dental papilla (Figure 5F, insert).

**Figure 6:**
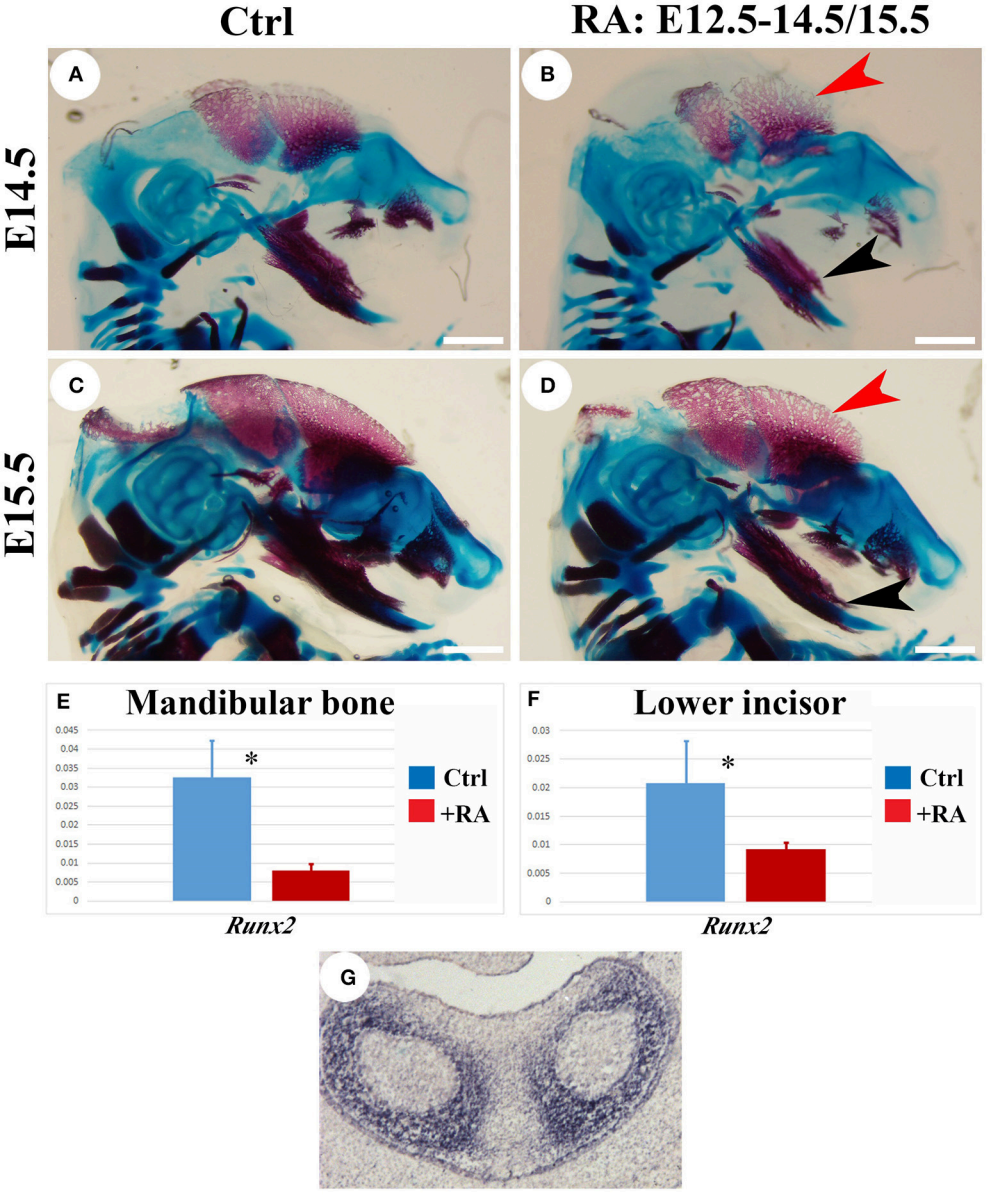
**Alizarin red/alcian blue analysis of E14.5 and E15.5 skeleton, RT-PCR and ***in situ*** hybridization analysis of ***Runx2*** expression in lower incisors and alveolar bone**. Osteoblast mineralization marked by red alizarin staining in untreated E14.5 **(A)** and E15.5 **(C)** heads is reduced in RA-treated **(B,D)** parietal bone (red arrowhead) and mandible (black arrowhead) at equivalent stages. Scale bars: 1 mm. RT-PCR analysis of *Runx2* mRNA at E14.5 shows significant reductions following RA treatments in mandibular bone **(E)** and lower incisor **(F)**. Target genes were normalized relative to *Gapdh*. Mean (*SD*). Student *t*-test: ^*^p < 0.05. **(G)** *In situ* hybridization detection of *Runx2* transcripts in E14.5 lower incisors (in absence of RA treatment).

Excesses of vitamin A or RA lead to skeletal fragility by reducing bone formation and by stimulating bone-resorbing osteoclasts (Henning et al., 2015). In the *Cyp26b1* mutant, impaired RA catabolism causes long-bone fusions and induces premature osteoblast differentiation into mineralizing osteocytes, truncating bone development in the craniofacial region (Maclean et al., 2009). Alizarin red/alcian blue staining of E14.5 skulls 2 days into RA regime revealed shortened mandibles, and truncated regions of ossified maxilla and premaxilla in the RA-treated animals (Figures 6A,B). The skull frontal plate was also smaller in treated animals, with less ossification. Malformed Meckel's cartilage and truncated mandibles could lead to incisor shortenings. Skeletal staining performed at E15.5 confirmed RA-reduced mineralization (Figures 6C,D). RT-PCR analysis showed reductions in *Runx2* mRNA in both lower incisor and adjacent alveolar bone at E14.5 in RA-treated animals (Figures 6E,F). A 3-fold reduction in the expression of this key target might account for reduced bone mineral deposition (Ducy and Karsenty, 1998), and its mesenchymal localization (Figure 6G) implies indirect enamel effects. To exclude systemic non-specific RA effects, we cultured isolated lower incisors or lower incisors with adjacent alveolar bone from E13.5 embryos (Figure S4). When 10^−8^ M RA was added to culture medium, *Enam* levels were dramatically reduced in isolated incisor cultures, indicating RA acted directly on tooth.

To compare global gene expression changes in control vs. RA-treated samples, E14.5 and E16.5 lower incisors were analyzed by high throughput RNA sequencing (RNA-seq). Principal component analysis (PCA plot, Figure S5) and scatter plots (Figure S6) revealed the overall changes observed at E14.5 were tightly clustered, statistically significant, yet often seen as net reductions of ~20–50% (Table 1). RNA-seq analysis confirmed broad effects of RA treatment affecting mineralization-inducing pathways, extracellular collagens, and calcium networks. Genes reduced in expression at E14.5 included *Runx, Dlx, Bmp*, and *Shh*, all known inducers of inner enamel epithelium differentiation (Bei, 2009). Net reduction in *Runx2* and *Dlx5* (Table 1) may act combinatorially to reduce the bone biomarker BGLAP (osteocalcin, (Hassan et al., 2004)), a target reduced by ~2-fold at E16.5 in RA-treated samples (Table 2). A novel RA-inhibited target is the matricellular protein Smoc2, whose mutation in human produces oligodontia, microdontia, abnormal root development, dentin dyplasia, and reduced alveolar bone growth (Bloch-Zupan et al., 2011). Retinoids are known to drive uncommitted progenitor cells into neuronal lineages (Maden, 2007). Consistently, DAVID (Database for Annotation, Visualization, and Integrated Discovery) analysis revealed a functional enrichment score of 1.4 E^−6^ for neuronal differentiation pathways. Increased *Neurogenin1* (*Neurog1*), *Neurogenin2* (*Neurog2*), and the notch target *Hes5*, are typical of an RA-induced neuronal differentiation profile (Table S2). At E16.5, *Enam* was the most reduced target (Table 2), also markedly reduced by *in situ* hybridization analysis (Figure S7). Reductions in *ameloblastin, X- linked amelogenin, amelotin*, and *kallikrein-related peptidase 4* (*Klk4*), all encoding enamel proteins (Núñez et al., 2015), were observed (Table 2). Notably, these reductions at E16.5 occur much earlier than the characterized times of action of the corresponding proteins in inductive, secretory, or maturation stages of rodent enamel development. Odontoblast-originating signals control ameloblast induction (Bei, 2009). Reductions in mineralization targets (*alkaline phosphatase*), odontoblast structural proteins (*dentin matrix protein 1, dentin sialophosphoprotein*), ossification biomarkers (*Bglap1/osteocalcin, Bglap2*), and calcium homeostasis pathways (*calcitonin, vitamin D receptor*) were all observed in E16.5 RA-treated samples. Table S3 summarizes how RA excess at E16.5 increases the expression of genes involved in retinoid signaling (including *Cyp26b1*), Wnt signaling, and neuronal differentiation.

**Table 1:** **Genes encoding regulators of bone growth, collagens, and proteins involved in calcium signaling and homeostasis, retinoid, and Shh pathways, reduced in expression in E14.5 RA-treated lower incisors**.

**Symbol**	**Name**	**FC log2**	***p*-value**
**BONE/OSTEOBLAST GROWTH**			
Bmp2	Bone morphogenetic protein 2	−0.35	1.08 E-02
Bmp3	Bone morphogenetic protein 3	−0.33	1.93 E-02
Bmp5	Bone morphogenetic protein 5	−0.43	1.72 E-02
Ostn	Osteocrin	−1.30	8,96 E-05
Sp7	Sp7 transcription factor 7	−0.80	2,62 E-05
Smoc2	SPARC modular calcium binding 2	−0.73	1,74 E-08
Runx2	Runt related transcription factor 2	−0.38	2.98 E-02
Runx3	Runt related transcription factor 3	−0.62	7.01 E-06
Dlx5	Distal-less homeobox 5	−0.58	1.64 E-03
Dlx3	Distal-less homeobox 3	−0.53	8.37 E-03
Mef2c	Myocyte enhancer factor 2C	−0.58	6.33 E-05
Ltbp2	Latent transforming growth factor β binding protein 2	−0.86	1.98 E-06
Fgf10	Fibroblast growth factor 10	−0.42	4.17 E-04
Fam20a	Family with sequence similarity 20A	−0.64	2.71 E-04
Tnn	Tenascin N	−1.18	2.01 E-10
Sox6	SRY (sex determining region Y)-box 6	−0.41	1.20 E-02
Tgfb3	Transforming growth factor β 3	−0.31	1.45 E-04
**COLLAGEN**			
Col1a1	Collagen, type I, α 1	−0.61	6.25 E-03
Col1a2	Collagen, type I, α 2	−0.43	3.62 E-02
Col5a1	Collagen, type V, α 1	−0.37	1,39 E-04
Col6a3	Collagen, type VI, α 3	−0.42	8.34 E-05
Col8a1	Collagen, type VIII, α 1	−0.79	3.74 E-04
Col8a2	Collagen, type VIII, α 2	−0.53	1.25 E-04
Col12a1	Collagen, type XII, α 1	−0.63	1.43 E-04
**CALCIUM SIGNALING AND HOMEOSTASIS**			
Capn3	Calpain 3	−1.03	1.78 E-03
Otof	Otoferlin	−0.82	3.68 E-03
Kcnma1	Potassium calcium-activated channel Mα 1	−0.54	8.47 E-03
Cacna2d2	Calcium channel, voltage dependent, α2/δ2	−0.48	2.26 E-03
**RETINOID SIGNALING**			
Aldh1a1	Retinaldehyde dehydrogenase 1	−0.80	1.22 E-02
Aldh1a3	Retinaldehyde dehydrogenase 3	−0.69	2.70 E-03
Rbp2	Retinol binding protein 2	−0.86	4.68 E-02
Cyp26c1	Cytochrome P450 26c1	−0.29	3.77 E-01
**SHH SIGNALING**			
Ihh	Indian hedgehog	−1.40	1.84 E-06
Hhip	Hedgehog-interacting protein	−0.65	1.64 E-05
Ptch1	Patched 1	−0.42	3.04 E-02

*Data are presented as log2 fold changes in RA-treated vs. control samples: for instance, a FC log2 value of −1.00 will correspond to a 50% mRNA level in the RA-treated samples*.

**Table 2:** **Summary of genes encoding enamel proteins, extracellular matrix components, proteins involved in bone growth pathways, or calcium and iron signaling/homeostasis, all reduced in expression according to RNA-seq analysis of E16.5 RA-treated lower incisors**.

**Symbol**	**Name**	**FC log2**	***p*-value**
**ENAMEL MATRIX PROTEINS**			
Enam	Enamelin	−3.47	4.99 E-013
Amtn	Amelotin	−1.85	1.53 E-004
Amelx	Amelogenin, X-linked	−1.7	1.01 E-004
Ambn	Ameloblastin	−1.42	1.78 E-003
**ENAMEL MODIFICATION**			
Klk4	Kallikrein peptidase 4	−1.62	1.14 E-003
Ppara	Peroxisome proliferator activated receptor α	−0.74	1.55 E-002
Fam20c	Family with sequence similarity 20, C	−0.66	7.31 E-003
**BONE GROWTH FACTOR PATHWAYS**			
Bglap	Bone gamma carboxyglutamate protein	−1.67	6.06 E-004
Bglap2	Bone gamma-carboxyglutamate protein 2	−1.15	2.41 E-002
Comp	Cartilage oligomeric matrix protein	−2.89	3.00 E-013
Phex	Phosphate endopeptidase X-linked	−1.01	4.10 E-002
Alpl	Alkaline phosphatase	−0.94	5.79 E-006
Col11a1	Collagen, type XI, α 1	−1.16	4.60 E-006
Col11a2	Collagen, type XI, α 2	−1.4	5.72 E-004
Col13a1	Collagen, type XIII, α 1	−1.34	7.25 E-008
**EXTRACELLULAR MATRIX**			
Dmp1	Dentin matrix protein 1	−0.94	7.47 E-002
Dspp	Dentin sialophosphoprotein	−0.85	9.79 E-002
Postn	Periostin	−0.56	1.39 E-004
**CALCIUM SIGNALING AND HOMEOSTASIS**			
Camk2a	Calcium/calmodulin protein kinase II α	−1.31	4.72 E-005
Camk2b	Calcium/calmodulin protein kinase II β	−1.41	1.33 E-005
Smoc2	SPARC related modular calcium binding 2	−0.51	5.02 E-004
Calcr	Calcitonin receptor	−2.91	1.22 E-014
Vdr	Vitamin D receptor	−1.45	6.81 E-008
Slc8a3	Solute carrier family 8 (sodium/calcium exchanger) 3	−1.17	3,80 E-003
Pth1r	Parathyroid hormone 1 receptor	−1.00	3.31 E-006
Ramp1	Receptor (calcitonin) activity modifying protein1	−1.09	1.00 E-006
**IRON HOMEOSTASIS**			
Hfe2	Hemochromatosis type 2	−1.04	3.70 E-002
Tfr2	Transferrin receptor 2	−0.99	1.34 E-003

*Data are presented as log2 fold changes (FC log2) in RA-treated vs control samples: for instance, a FC log2 value of −1.00 will correspond to a 50% mRNA level in the RA-treated samples. Adjusted p-value takes into account a false discovery rate wherein 5% of samples with a p-value of 0.050 will result in false positives*.

## Discussion

### RA excess affects enamel matrix protein expression prior to ameloblast differentiation

AI refers to rare, inherited diseases characterized by a defect in enamel formation with clinical heterogeneity even within the same family (Bloch-Zupan et al., 2012). These variations, also observed in acquired enamel defects, have been proposed to be due to environmental excess in fluoride (Yang et al., 2015), or to other nutritional, environmental, or behavioral changes (Li et al., 2013), along with genetic makeup of an individual. Retinoids are regulators of skeletal growth and patterning, known to lead to skeletal fragility when given in excess (Henning et al., 2015). Since reciprocal interactions between enamel organ and ectomesenchyme are necessary for alveolar bone, periodontal, and tooth differentiation, we examined if nutritional RA excess could have additional effects on developing teeth. Prior to this study, little was known about *in vivo* effects of RA on enamel cytodifferentiation. Here we find that RA treatment at a defined window of murine development resulted in permanent defects resembling human AI. Ameloblast differentiation begins at the advanced bell stage (~E18.5 in mouse), when the inner enamel epithelial originating cells express enamel secretory proteins and follow by processing enzymes (Bei, 2009; Bloch-Zupan et al., 2012). Assuming normal rodent nocturnal feed, in our experimental protocol RA exposure would begin at the bud stage of tooth formation (E13.0). We observed that at E14.5, RA excess impairs expression of *Runx, Dlx*, bone morphogenetic proteins, while levels of enamel secretory proteins are not altered in either our RNA-seq analysis or RT-PCR analysis at these same stages (data not shown). RA excess changes likely occur prior to ameloblast differentiation, with molecular alterations indicating effects on pro-mineralization signaling.

### Retinoids have early targets in mineralized tissue, and later effects on enamel proteins

**Figure 7:**
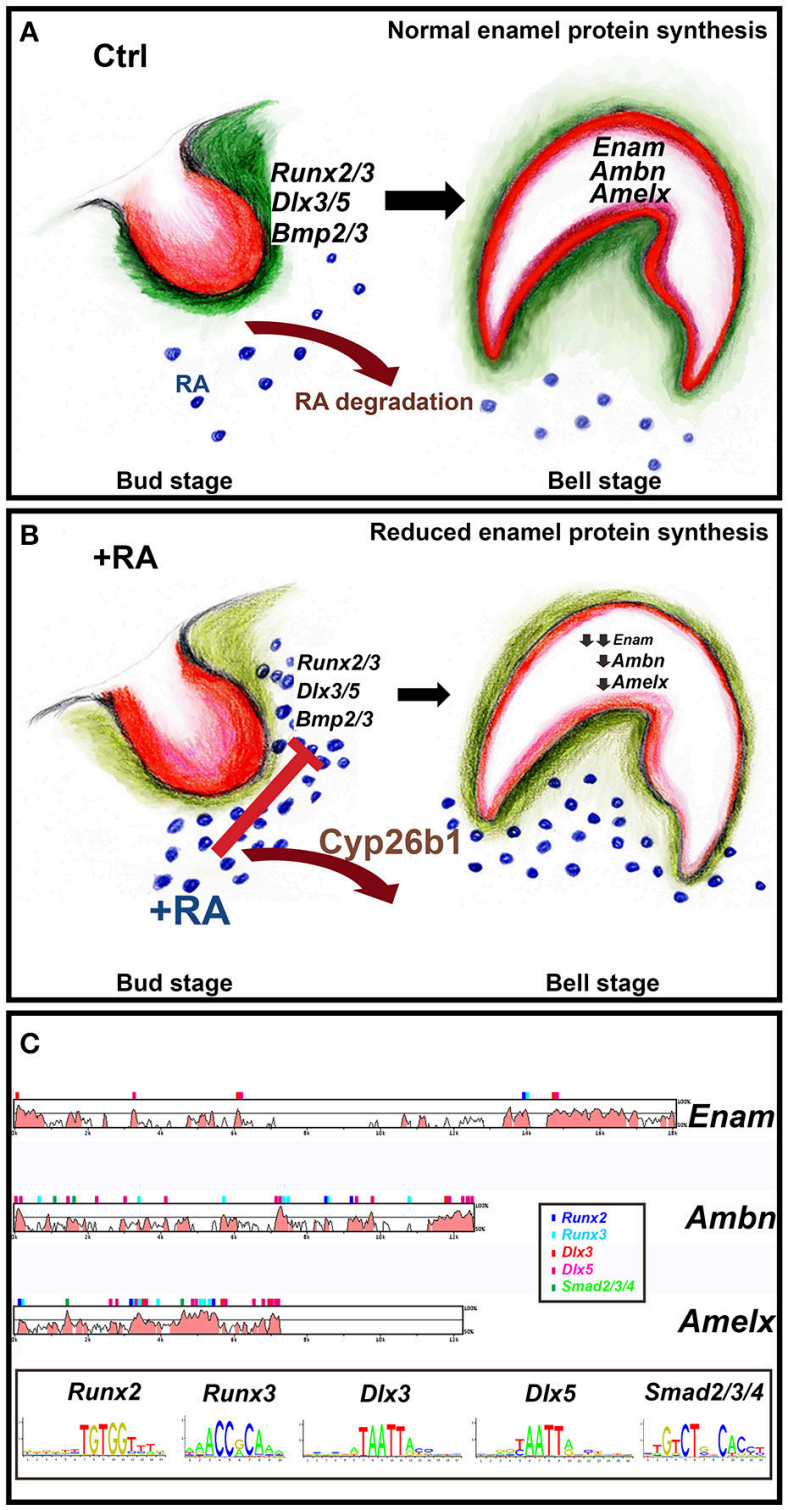
**Model of how RA excess impairs enamel formation**. Dental mesenchyme (green) and oral epithelium (red) have reciprocal interactions during tooth development (Balic and Thesleff, 2015) **(A,B)**. **(A)** Normally the entire developing tooth is shielded from RA by actions of Cyp26 RA-degrading enzymes. This allows proper expression of *Runx2/3, Dlx3/5*, and *Bmp2/3* in the mesenchyme condensing next to the oral epithelium at the bud stage **(B)**. These targets are reduced by excess RA. *Enam, Ambn*, and *Amelx* exhibit strong reductions at the late bell stage, impairing enamel crystallization **(C)**. The binding site motifs of *Runx2/3, Dlx3/5*, and *Smad2/3/4* were obtained from the JASPAR database (http://jaspar.genereg.net) and used to find potential binding sites in conserved regions using the rVISTA database (http://genome.lbl.gov/vista/index.shtml).

Enamel-reducing effects of RA supplementation at E13–14.5 coincide with the initial stages of intramembraneous ossification. An early target is *Runx2*, a master regulator of skeletal mineralization. *Runx2*^−/−^ mouse mutants have a block in endochondral and intramembraneous osteoblast maturation, and site-specific reductions in collagen type I and alkaline phosphatase expression (Ducy and Karsenty, 1998). *Runx2*^−/−^ mutants lack differentiated odontoblast and ameloblast matrices, indicative of late bell-stage defects (D'Souza et al., 1999; Bronckers et al., 2001). We observe RA-dependent *Runx2* reductions at E14.5 bud stage incisors, but not at E16.5, suggesting earlier effects. Cleidocranial dysplasia, an autosomal dominant condition caused by mutations of *RUNX2*, likewise results in insufficient dentin and enamel mineralization (Xuan et al., 2010) and other dental anomalies (Camilleri and McDonald, 2006). Terminal ameloblast differentiation requires odontoblast-originating signals and matrix components (Balic and Thesleff, 2015). Reports of evolutionarily conserved Runx2-binding sites in *Enam, Ambn*, and *Odam* gene promoters Dhamija and Krebsbach, 2001; Lee et al., 2010, suggested a model of RA-inhibitory effects (Figure 7). Retinoid excess at E14.5 would induce relatively small, yet combined reductions in *Runx2/3, Dlx3*/5, and *Bmp2*/*3*, predominantly mesenchymal targets that surround the bud stage tooth (Figure S8). By E16.5 these factors collectively regulate early epithelial (enamel organ) expression of *Enam, Ambn*, and *Amelx*. This is plausible because these enamel targets possess evolutionarily conserved binding site-motifs in their proximal promoters (Loots et al., 2002; Cartharius et al., 2005; Figure 7C; Table S4). While the bell stage tooth reacts to the high retinoid environment by up-regulating *Cyp26b1* levels (Table S3), this change is insufficient to offset strong reductions in enamel matrix protein secretion, which then induce ameloblast differentiation defects.

### Many enamel targets are in the secretory phosphoprotein-encoding (SCPP) gene cluster

Our RNA-seq analysis on the whole lower incisors uncovered many genes significantly reduced at E16.5 that regulate ameloblast differentiation, enamel formation, and dentin/bone development (Table 2). Hence combinatorial deficits in enamel secretory protein expression included reductions in X-linked amelogenin (*AMELX*, OMIM: 300391) (Gibson et al., 2001; Barron et al., 2010), *AMBN* (OMIM: 601259) (Fukumoto et al., 2004), *AMTN* (OMIM: 610912) (Nakayama et al., 2015), along with *Odam* (OMIM: 614843). These genes belong to the evolutionarily-related SCPP gene cluster, a linked group of genes also containing members regulating skeletal mineralization (Kawasaki and Weiss, 2008). These SCPP enamel proteins contain structural domains promoting calcium sequestration and regulating crystal adsorption (Addison and McKee, 2010), and their mutations produce hypomineralized enamel phenotypes including altered prism patterning and increased cellular apoptosis in both patients and mouse models (Fukumoto et al., 2004; Bei, 2009; Hu et al., 2014; Núñez et al., 2015). Reductions in the SCPP cluster appeared specific to enamel expression. Other enamel-regulating targets included the kallikrein-related peptidase 4, essential for removing enamel proteins and bio-mineralization (OMIM: 603767), the peroxisome proliferator-activated receptor (PPAR) alpha, required to achieve normal enamel mineralization (OMIM: 170998) (Sehic et al., 2009), and FAM20C, whose mutation produces severe enamel defects in human and mouse (Wang et al., 2012; Acevedo et al., 2015).

### RA excess alters the osteoblast, odontoblast, and ameloblast lineages

Over 80 years ago, severe nutritional vitamin A deficiency was reported to reduce enamel formation (Mellanby, 1941), but since this time no genetic models of RA deficiency have been reported with ameloblast or other primary tooth defects. No defects are observed in mouse mutants for the RA-synthesizing enzymes Raldh2 and Raldh3 (Dupé et al., 2003, our unpublished observations), implicating a more severe RA deficiency is required. More likely vitamin A intake levels are usually quite high, hence cellular RA levels need to be reduced during ossification. Both hypervitaminosis A and very low serum retinol levels produce skeletal fragility, poor bone health, and osteoporosis risk (Henning et al., 2015; Green et al., 2016). During mineralization, site-specific increases in CYP26 enzymes are required for bone formation (Minegishi et al., 2014). *Cyp26b1*^−/−^ mutants have truncated, under-ossified mandibles, possibly due to RA excess perturbing neural crest migration, or alternatively to defects in osteoblast maturation. Incisor defects, while noted, were not characterized (Maclean et al., 2009). Human mutations (both null and hypomorphic) for this RA-catabolizing enzyme produce calvarial hypoplasia and craniosynostosis (Laue et al., 2011). In our experiments, *Cyp26b1* is potently induced in RA-treated E16.5 incisors (Table S3). Even so, enamel defects are still observed.

The dentino-alveolar bone complex regulates tooth development. We observe rapid *in vivo* effects of RA reducing *Runx2*, and its collagenous and mineralization targets. This rapid rodent response to hypervitaminosis A (Lind et al., 2013) is similar to effects in humans (Henning et al., 2015). These RA excesses target enamel matrix protein production. Phenotypic differences in AI severity have been described between family members with identical mutations (see Prasad et al., 2016, for a recent list). Affected patients could be sensitized to otherwise benign alteration in vitamin consumption, RA catabolism pathways, or defects in the tooth and bone biosynthesis programs. Accumulating mutations might sensitize fetal development to environmental factors, including nutrition, explaining variability in AI morphogenetic phenotypes. Similar models have been proposed for RA interactions with the DiGeorge/chromosome 22q1 deletion syndrome (Maynard et al., 2013). Even physiological RA excesses, in the context of additional genetic alterations (which otherwise would produce benign changes) could have net consequences contributing to clinical variations in oral manifestations of rare diseases.

## Author contributions

Study design: SM, AB, and KN. Data collection: SM, VL, EM, BS, and KN. Data analysis: SM, VL, JH, AB, and KN. Drafting manuscript: SM, PD, AB, and KN. Revising manuscript content: SM, JH, PD, AB, and KN. Approving final version of manuscript: SM, VL, JH, EM, BS, PD, AB, and KN. KN takes responsibility for the integrity of the data analysis.

## Funding

This work was supported by a grant from the University Hospital of Strasbourg (API, 2009–2012, “Development of the oral cavity: from gene to clinical phenotype in Human”), the EU-funded projects (ERDF) A27 “Oro-dental manifestations of rare diseases” in the framework of the RMT-TMO Offensive Sciences initiative INTERREG IV and INTERREG V No. 1.7 RARENET, the Institut d'Etudes Avancées (Institute of Advanced Studies) de l'Université de Strasbourg (USIAS Fellows 2015), and the grant ANR-10-LABX-0030-INRT managed by the Agence Nationale de la Recherche under the frame program Investissements d'Avenir ANR-10-IDEX-0002-02. Sequencing was performed by the IGBMC Microarray and Sequencing platform, supported by the France Genomics National Infrastructure, funded as part of the Investissements d'Avenir program (ANR-10-INBS-0009).

### Conflict of interest statement

The authors declare that the research was conducted in the absence of any commercial or financial relationships that could be construed as a potential conflict of interest. The handling Editor declared a past co-authorship with the authors VL and AB and states that the process nevertheless met the standards of a fair and objective review.
