# Supplementary material for: Retinoic Acid Excess Impairs Amelogenesis Inducing Enamel Defects
Source: Front Physiol. 2017 Jan 6;7:673. doi: 10.3389/fphys.2016.00673 (PMC5217128; doi:10.3389/fphys.2016.00673)
Supplement: Supplementary file 1 [file DataSheet1.DOCX]

Supplementary Material

**Retinoic acid excess impairs amelogenesis inducing enamel defects**

# Supplementary Text

**Methods:**

**Incisor explant culture**:

Pregnant CD1 mice were sacrificed at E13.5, and paired lower incisors (still bearing cartilage connection) were dissected, then cultured for 7 days on Costar 6-well Transwell dishes containing 1.5 mL of Advanced Dulbecco’s Modified Eagle Medium F12 (DMEM F12 -GIBCO-BRL) containing 10% fetal calf serum, 1% penicillin-streptomycin (Sigma), and 20 µg/mL ascorbic acid (Sigma). All-trans retinoic acid (Sigma) at a concentration of 5 mg/mL in ethanol was diluted in media and added to cultures. Cultures were shielded from light and incubated at 37^°^C in 5% CO_2_, and medium was changed every two days.

**Histological analysis**

Heads of 7 day-old control and RA-treated mice were fixed in 4% paraformaldehyde, rinsed, demineralized in 10% EDTA at room temperature for 8 days (the demineralizing solution was changed every two days). After thorough water washes, the heads were dehydrated in a graded ethanol series, cleared in Histosol, and embedded in paraffin at 60°C. Sagittal sections (8 µm) were stained with hematoxylin/eosin. A detailed histology protocol can be found at <http://www.empress.har.mrc.ac.uk> phenotyping, Necropsy Exam, Pathology, Histology section.

**RNA sequencing**

The mRNAseq libraries were prepared according to a modified Illumina protocol. Purified mRNA was isolated from total RNA using oligo-dT magnetic beads, and fragmented using divalent cations (95°C for 5 minutes). mRNA fragments were reverse transcribed using random primers, and second strand cDNA synthesis was performed. The cDNA fragments were blunt-ended, phosphorylated, and ligated to single-ended adapter dimers. Following PCR amplification, excess PCR primers and dimer adapters were removed using AMPure bead purification (Agencourt Biosciences Corporation). Size selection by electrophoresis in agarose gel was used to isolate DNA fragments of ~250-350 bp. These were excised and purified using QIAquick Gel Extraction Kit (Qiagen). DNA library quality and quantification was assessed using a 2100 Bioanalyzer (Agilent). The libraries were loaded in the flowcell (at a 6 picomolar concentration) and sequenced using Illumina Genome Analyzer IIx equipment which performed single-end 54 base reads. Image analysis and base calling were performed with the 1.6 Illumina Pipeline.

***In silico* analysis of the enamel matrix protein regulatory regions**

For *in silico* comparative genomic analysis of the *Enam*, *Ambn*, and *Amelx* regulatory regions we used a combination of web-based programs. Blast searches were conducted using the National Center for Biotechnology Information programs (<https://www.ncbi.nlm.nih.gov/>). We used the whole human and mouse sequences of each gene to find evolutionarily conserved regions. Vista plot analysis was performed using the mVista program at <http://genome.lbl.gov/vista/mvista/submit.shtml>. To identify binding element motifs of the *Runx2*/*3*, *Dlx3*/*5*, and *Smad2/3/4* transcription factors we used the web-based program JASPAR (version 2016) at <http://jaspar.genereg.net>. To find transcription factor binding sites at the conserved regions both rVista and (<http://genome.lbl.gov/vista/rvista/submit.shtml> and MatInspector (<https://www.genomatix.de/online_help/help_matinspector/matinspector_help.html#seq_selection>) programs were used. Gene expression databases (<http://bite-it.helsinki.fi>) and (<http://genepaint.org>) were used to verify expression domains of transcription factors potentially binding to the selected enamel matrix proteins.

# Supplementary Figures and Tables

## Supplementary Figures


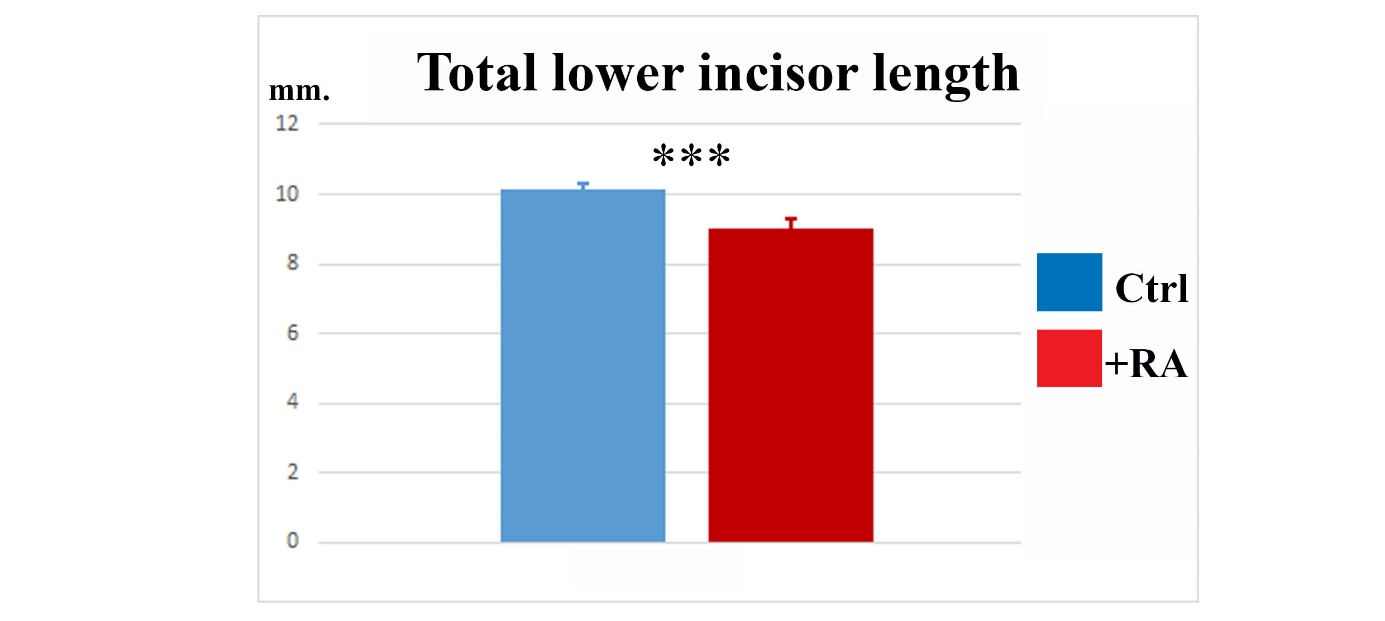


**Figure S1. Mean values of total lower incisor length calculated by micro-CT analysis.** Incisor length was measured form the most posterior to the tip of the incisal edge of lower incisors. In RA-treated samples, total lower incisor length is reduced by ~10% (*** *p*<0.001).

**
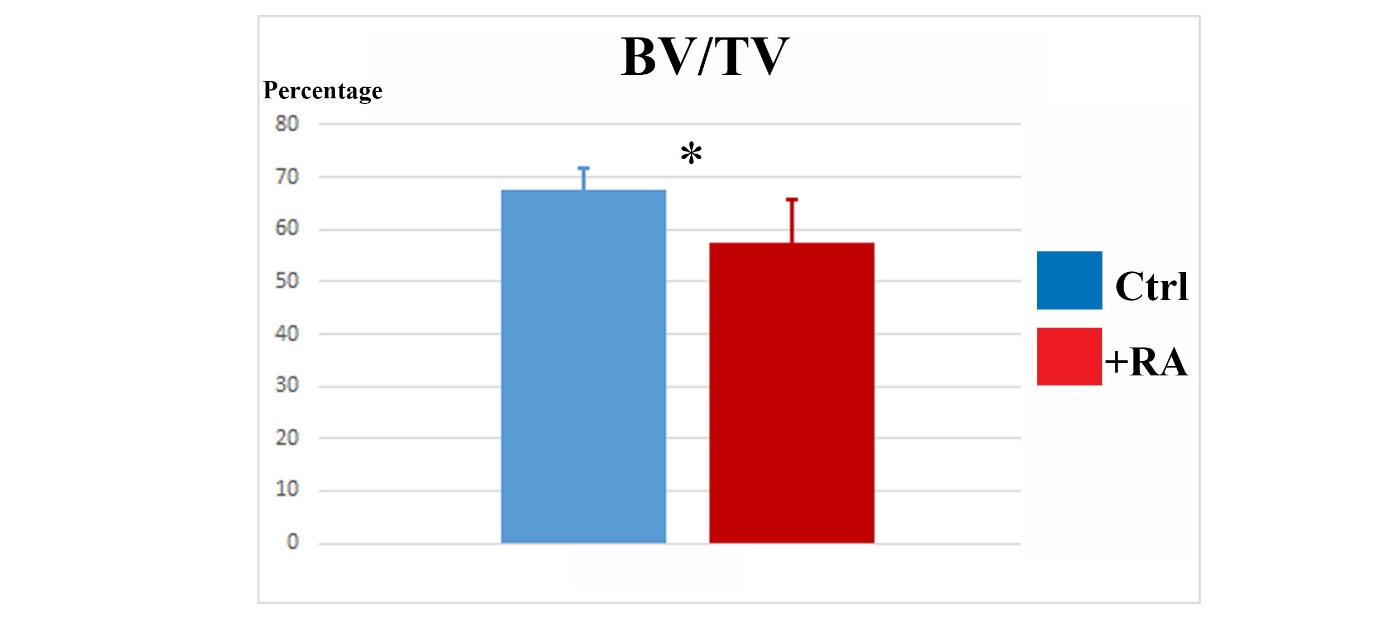
**

**Figure S2. Mean values of bone volume/tissue volume (BV/TV) analyzed by micro-CT analysis.** Percentage of alveolar bone around the lower first molar divided by total volume. In RA-treated samples, BV/TV is significantly reduced. (* *p*<0.05).


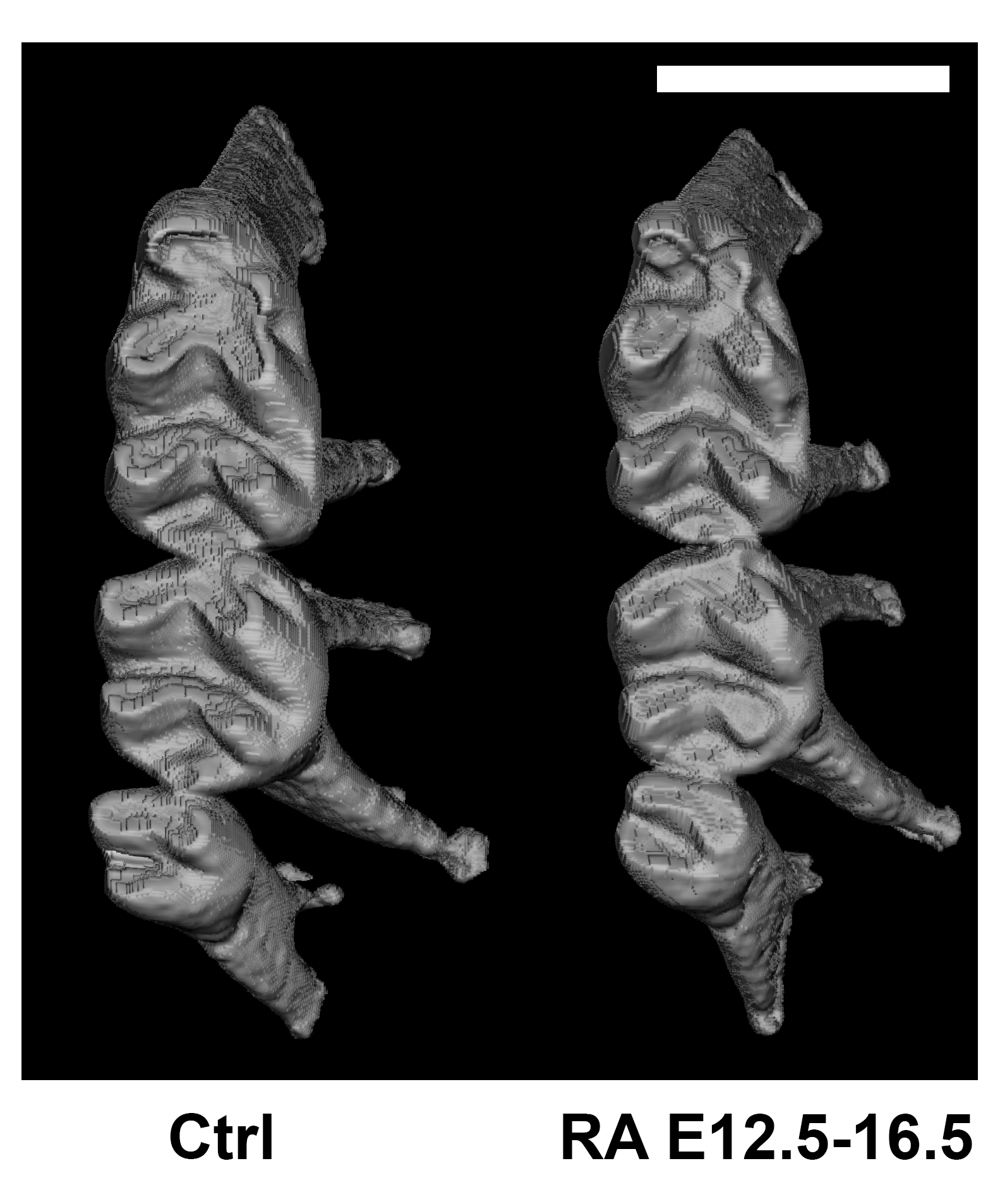


**Figure S3. Micro-CT imaging comparing lower molars from 50 day-old mice: untreated (left side) and following E12.5-16.5 RA treatment (right side).** Overall, the number of cusps, root morphology, and overall appearance is similar, although moderate growth reductions are observed. Scale bar: 1 mm.


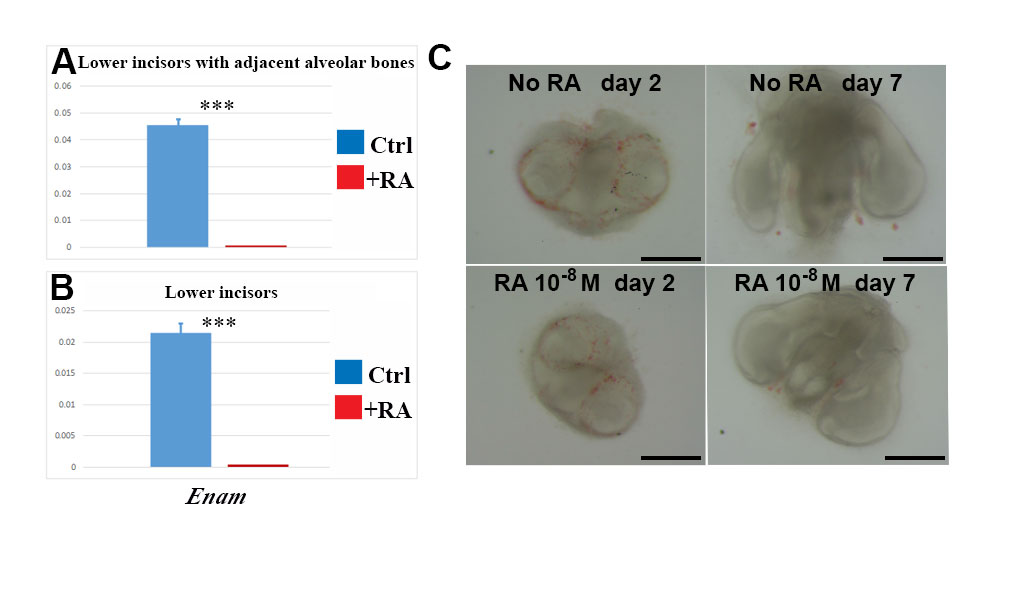


**Figure S4. RT-PCR analysis of *Enam* transcripts after Transwell explant culture of E13.5 lower incisors for 7 days.** Panel A shows effects of 10^-8^ M RA when incisors are cultured with adjacent alveolar bone. Panel B shows similar effects of RA in reducing *Enam* in isolated incisors (*** *p*<0.001). Photos of incisor explants in culture are shown in C. Note equivalent growth of incisors during RA treatment compared with control untreated samples. Scale bars: 500 µm.


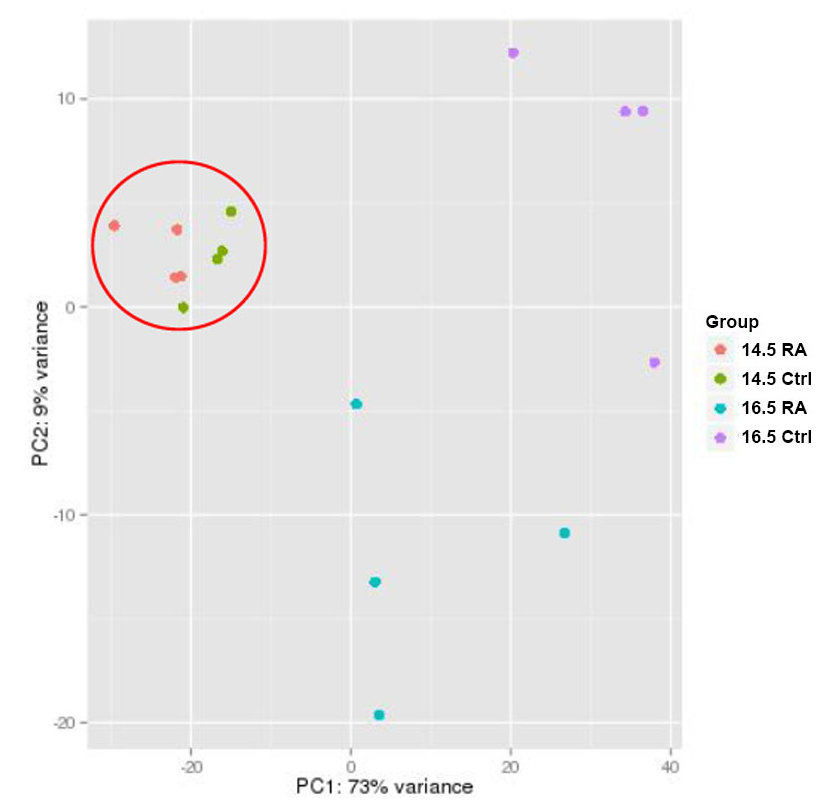


**Figure S5. Principal component analysis (PCA plot) visualizing overall effect of retinoid treatments on the incisor RNA transcriptome.** At E14.5, RA-treated and control samples show tight clustering. At E16.5 larger expression changes and sample disparity are observed.


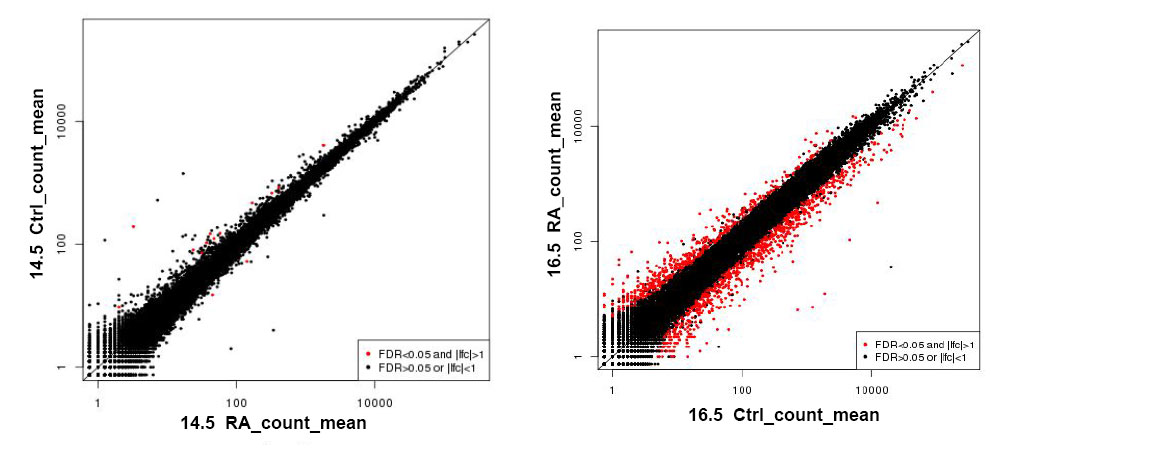


**Figure S6. Comparison between E14.5 untreated and RA-treated samples (left), and E16.5 untreated and RA-treated samples (right) using scatterplot diagrams** (obtained with DESeq2). At E14.5, less than 20 genes had fold-changes (log2) > 1 (red dots). At E16.5, using a false discovery rate (FDR)<0.05 and log2 fold-change > 1, the total number of significantly differentially expressed genes is 1375. The number of overexpressed genes is 502, whereas the number of underexpressed genes is 873.

**
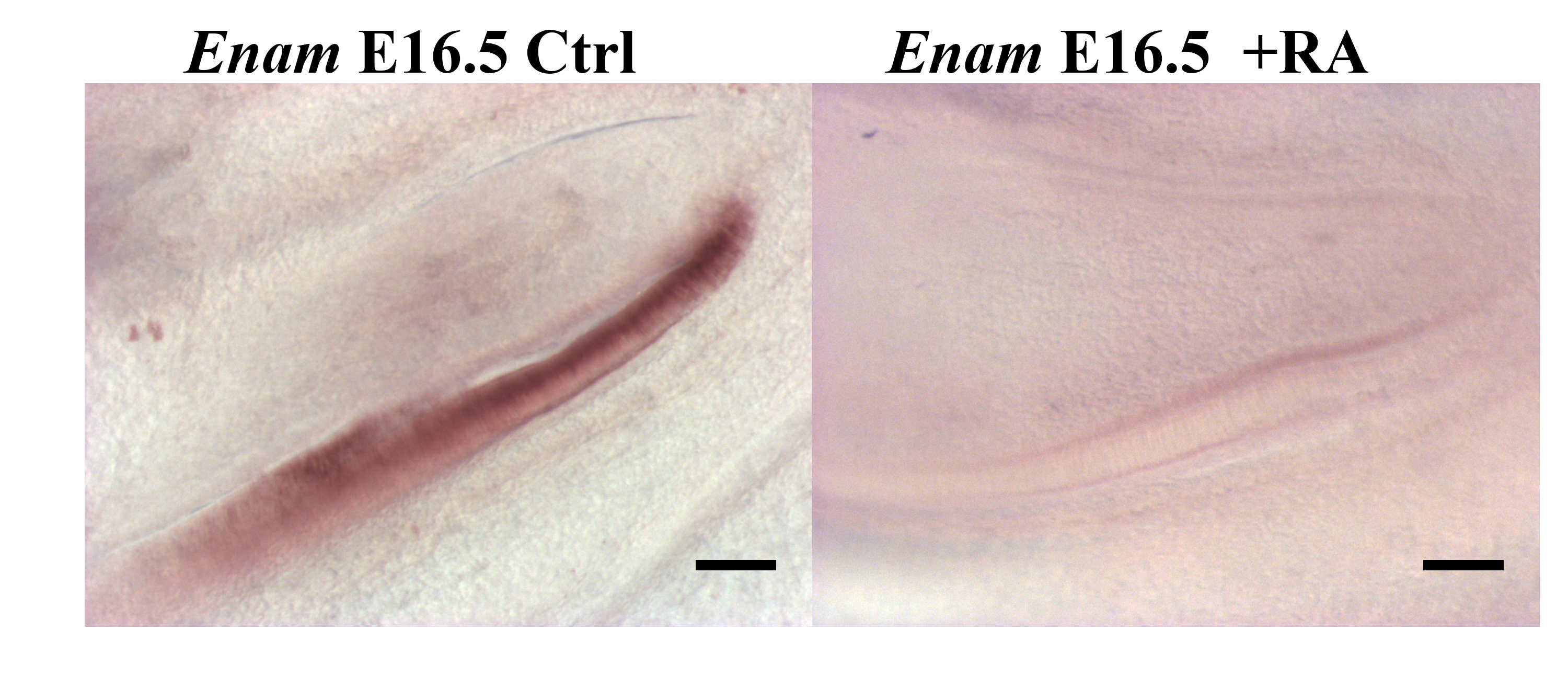
**

**Figure S7. In situ hybridization localization of *Enam* in the lower incisor pre-secretory ameloblasts at E16.5**. Marked expression of *Enam* is detected in pre-secretory stage ameloblasts. In RA-treated samples, *Enam* is significantly reduced. Scale bars: 100 µm.

**
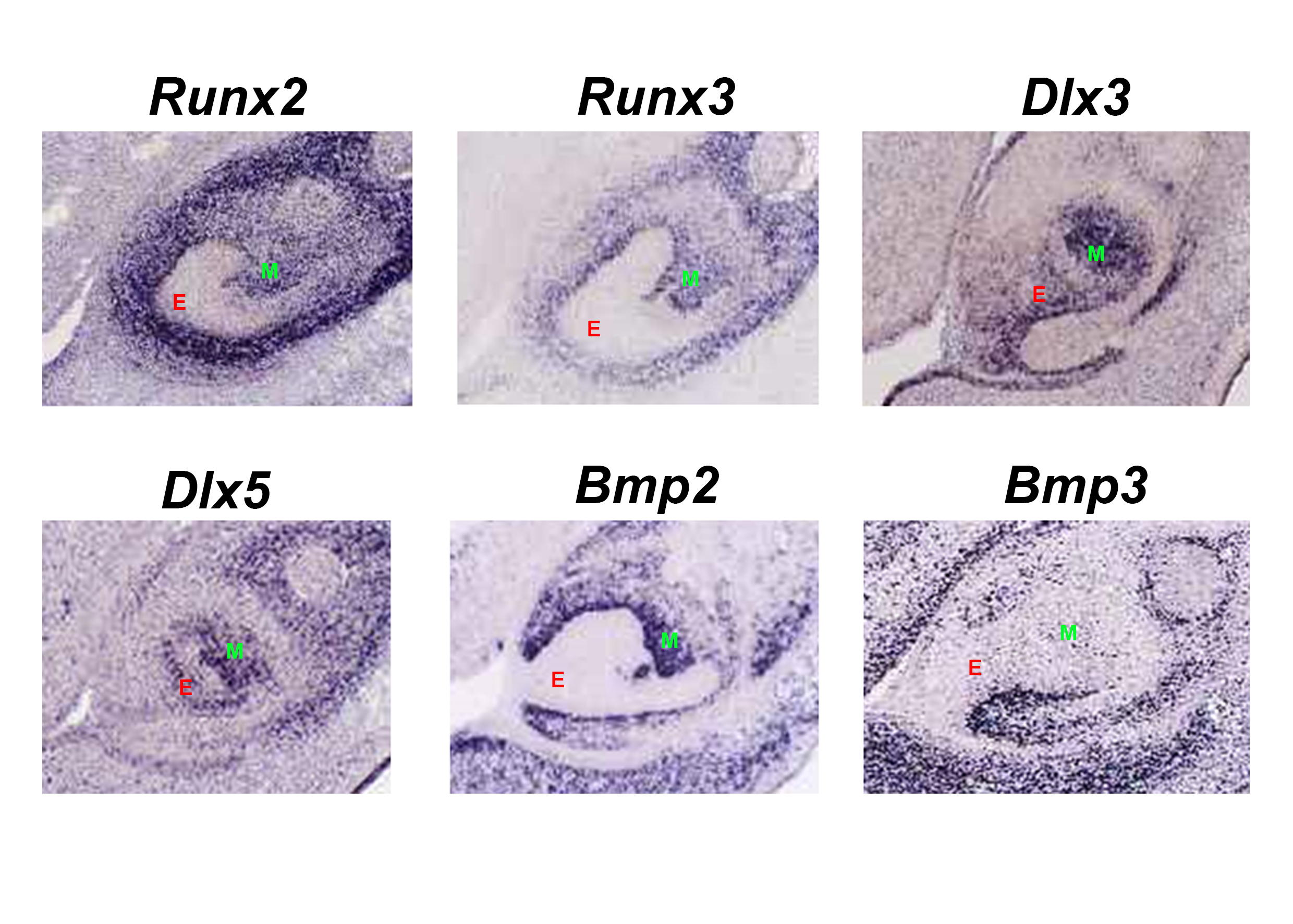
**

**Figure S8. In situ hybridization localization of *Runx2, Runx3, Dlx3, Dlx5, Bmp2,* and *Bmp3* in lower incisors at E14.5**. Marked expression of these genes is detected predominantly in dental mesenchymal tissue. All images are obtained from the Genepaint database (<http://genepaint.org/>). Abbreviations: epithelium (E), mesenchyme (M).

## Supplementary Tables

| NAME | Forward Primer | Reverse Primer |
| --- | --- | --- |
| *Enam* | TGC AGA AAT CCG ACT TCT CCT | CAT CTG GAA TGG CAT GGC A |
| *Ambn* | ATG AAG GGC CTG ATC CTG TTC | GTC TCA TTG TCT CAA GGC TCA AA |
| *Odam* | AGC TTT GCT GGA CTA TTC CCA | GTA CCA CAT AGG ACA TAG GGC T |
| *Runx2* | AGA GTC AGA TTA CAG ATC CCA GG | TGG CTC TTC TTA CTG AGA GAG G |
| *Bmp2* | GGG ACC CGC TGT CTT CTA GT | TCA ACT CAA ATT CGC TGA GGA C |
| *Dlx3* | CAC TGA CCT GGG CTA TTA CAG C | GAG ATT GAA CTG GTG GTG GTA G |
| *Dlx5* | TCT CTA GGA CTG ACG CAA ACA | GTT ACA CGC CAT AGG GTC GC |
| *Ihh* | CTC AGA CCG TGA CCG AAA TAA G | CCT TGG ACT CGT AAT ACA CCC AG |
| *Gapdh* | AGG TCG TGT TGA ACG GAT TTG | TGT AGA CCA TGT AGT TGA GGT CA |

**Table S1**: Primer sequences used for real-time RT-PCR analysis.

| Symbol | Name | FC log2 | *p*-value |
| --- | --- | --- | --- |
| Retinoid signaling | | | |
| Rarb | retinoic acid receptor β | 0.50 | 1.29 E-02 |
| Retsat | retinol saturase (all-trans-retinol 13,14 reductase) | 0.40 | 2.42 E-03 |
| Rdh16 | retinol dehydrogenase 16 | 0.32 | 2.38 E-01 |
| Neuronal transcription pathway | | | |
| En2 | engrailed 2 | 1.96 | 4.63 E-06 |
| Neurog1 | neurogenin 1 | 0.96 | 2.58 E-03 |
| Neurog2 | neurogenin 2 | 1.29 | 1.22 E-03 |
| Sox1 | SRY (sex determining region Y)-box 1 | 1.09 | 7.94 E-03 |
| Hes5 | hairy and enhancer of split 5 | 0.99 | 4.07 E-03 |
| Dbx1 | developing brain homeobox 1 | 0.93 | 2.38 E-02 |
| Neurod4 | neurogenic differentiation 4 | 0.86 | 2.19 E-02 |
| Neurod1 | neurogenic differentiation 1 | 0.83 | 4.11 E-02 |
| Otx2 | orthodenticle homolog 2 | 1.45 | 9.09 E-04 |
| Cell death | | | |
| Prok1 | prokineticin 1 | 1.20 | 5.98 E-03 |
| Prokr2 | prokineticin receptor 2 | 0.88 | 2.76 E-03 |
| Hrk | harakiri, BCL2 interacting protein | 0.76 | 1.50 E-02 |
| Blood lineage | | | |
| Hbq1a | hemoglobin, theta 1A | 0.87 | 3.52 E-02 |
| Vwf | Von Willebrand factor homolog | 0.73 | 2.96 E-06 |
| Notch signaling | | | |
| Hey2 | hairy/enhancer-of-split with YRPW motif 2 | 0.57 | 3.65 E-02 |
| Dtx3 | deltex 3 homolog | 0.33 | 1.40 E-03 |

**Table S2**: Summary of genes increased in expression in E14.5 RA-treated lower incisors. Selected genes are members of the retinoic acid pathway, transcriptional regulators of neuronal development, or related to cell death, blood lineage commitment, or Notch signaling. Data are presented as log2 fold changes in RA-treated versus control samples: for instance, a FC log2 value of 1.00 will correspond to a 2-fold mRNA increase in the RA-treated samples.

| Symbol | Name | FC log2 | *p*-value |
| --- | --- | --- | --- |
| Retinoic acid | | | |
| Cyp26b1 | cytochrome P450 26b1 | 2.26 | 2.66 E-023 |
| Rarb | retinoic acid receptor β | 1.16 | 2.55 E-007 |
| Stra6 | stimulated by retinoic acid gene 6 | 1.09 | 4.11 E-004 |
| Wnt signaling | | | |
| Wnt4 | wingless-type MMTV integration site 4 | 0.94 | 4.33 E-008 |
| Wnt5a | wingless-type MMTV integration site 5 α | 0.96 | 4.89 E-014 |
| Wnt5b | wingless-type MMTV integration site 5 β | 0.42 | 3.72 E-002 |
| Wnt7b | wingless-type MMTV integration site 7 β | 0.94 | 1.36 E-006 |
| Wnt11 | wingless-type MMTV integration site family 11 | 0,59 | 3.04 E-004 |
| Wisp3 | WNT1 inducible signaling 3 | 0.8 | 4.36 E-002 |
| Fzd10 | frizzled homolog 10 | 1.13 | 1.40 E-007 |
| Neuronal differentiated | | | |
| Sycp2l | synaptonemal complex protein 2 | 2.58 | 2.10 E-008 |
| Cntn2 | contactin 2 | 1.68 | 9.05 E-010 |
| Fscn2 | fascin homolog 2, actin-bundling protein | 1.65 | 9.15 E-005 |
| Gldn | gliomedin | 1.57 | 4.54 E-007 |
| Nps | neuropeptide S | 1.51 | 1.36 E-003 |
| Syt16 | synaptotagmin XVI | 1.48 | 2.08 E-008 |
| Cacna2d3 | calcium channel, voltage-dependent, α2/δ3 | 1.48 | 1.26 E-008 |
| Kcnmb2 | potassium large conductance channel M β 2 | 1.47 | 7.02 E-004 |
| Mc5r | melanocortin 5 receptor | 1.14 | 7.11 E-004 |
| Scg2 | secretogranin II | 1.13 | 6.06 E-003 |
| Grid2 | glutamate receptor δ 2 | 1.12 | 3.03 E-006 |
| Stx19 | syntaxin 19 | 1.11 | 6.45 E-004 |
| Gabrg3 | gamma-aminobutyric acid (GABA) A receptor, γ3 | 1.11 | 9.48 E-005 |
| Gprin2 | G protein regulated inducer of neurite outgrowth 2 | 1.08 | 3.94 E-003 |

**Table S3**: Genes of the retinoic acid pathway, or involved in Wnt signaling or neuronal differentiation, increased in expression in E16.5 RA-treated lower incisors. Data are presented as log2 fold changes in RA-treated versus control samples.

| Seq. name | Matrix Family | Detailed Matrix info. | Position | Core sim. | Matrix sim. | Evidence | Sequence |
| --- | --- | --- | --- | --- | --- | --- | --- |
| GXP_152236  Enam | V$DLXF | DLX-1, -2, and -5 binding sites | 95-113 | 1 | 0.982 | V$DLXF TF: Dlx3 | attccagtttAATTacgta |
|  | V$DLXF | DLX-1, -2, and -5 binding sites | 100-118 | 1 | 0.976 | V$DLXF TF: Dlx3 | gttaatacgtAATTaaact |
|  | V$HAML | RUNX3, AML2 | 798-812 | 1 | 0.971 | V$HAML TF: Runx2 | attTGTGgtgtttct |
| GXP_5974669  Enam | V$HAML | RUNX3, AML2 | 57-71 | 1 | 0.971 | V$HAML TF: Runx2 | attTGTGgtgtttct |
| GXP_152087  Ambn | V$DLXF | Distal-less homeobox 2 | 71-89 | 1 | 0.937 | V$DLXF TF: Dlx3 | aacacacagtAATTgtgtc |
|  | V$DLXF | Distal-less homeobox 5 | 76-94 | 1 | 0.925 | V$DLXF TF: Dlx3 | tcagtgacacAATTactgt |
|  | V$HAML | RUNX3, AML2 | 381-395 | 1 | 0.947 | V$HAML TF: Runx2 | ggaTGTGgtcattgg |
| GXP_152088  Ambn | V$DLXF | Distal-less homeobox 3 | 45-63 | 1 | 0.933 | V$DLXF TF: Dlx3 | ccctagagaTAATttggtg |
|  | V$DLXF | Distal-less 3 homeo-domain TF | 206-224 | 1 | 0.912 | V$DLXF TF: Dlx3 | caaaacagaTAATggcttg |
|  | V$DLXF | Distal-less 3 homeo-domain TF | 402-420 | 1 | 0.985 | V$DLXF TF: Dlx3 | ggtgtggacTAATtgcagg |
|  | V$DLXF | Distal-less homeobox 2 | 407-425 | 1 | 0.928 | V$DLXF TF: Dlx3 | gtgctcctgcAATTagtcc |
| GXP_229912  Amelx | V$HAML | Runx2/ CBFA1 | 36-50 | 1 | 0.841 | V$HAML TF: Runx2 | acagGTGGttttcta |
|  | V$DLXF | Distal-less 3 homeo-domain TF | 266-284 | 1 | 0.992 | V$DLXF TF: Dlx3 | aagtaacgtTAATtgctag |
|  | V$DLXF | Distal-less homeobox 2 | 271-289 | 1 | 0.924 | V$DLXF TF: Dlx3 | cagttctagcAATTaacgt |
|  | V$HAML | AML1/CBFA2 Runt domain | 667-681 | 1 | 0.991 | V$HAML TF: Runx2 | cactGTGGtcatttc |
| GXP_152163  Amtn | V$DLXF | Distal-less homeobox 3 | 292-310 | 1 | 0.884 | 0 | atgcctatatAATTttaaa |

**Table S4**: **Potential transcription factor binding sites (TFBS) for *Enam*, *Ambn*, *Amelx*, and *Amtn* with *Dlx* and *Runx* families.** These results were generated by MatInspector (<https://www.genomatix.de/online_help/help_matinspector/matinspector_help.html>).
